# Microporous organic nanotube assisted design of high performance nanofiltration membranes

**DOI:** 10.1038/s41467-022-35681-9

**Published:** 2022-12-27

**Authors:** Shuangqiao Han, Junyong Zhu, Adam A. Uliana, Dongyang Li, Yatao Zhang, Lin Zhang, Yong Wang, Tao He, Menachem Elimelech

**Affiliations:** 1 School of Chemical Engineering, Zhengzhou University, Zhengzhou, 450001 China; 2 Department of Chemical and Biomolecular Engineering, University of California, Berkeley, CA 94720 USA; 3 Key Laboratory of Biomass Chemical Engineering, College of Chemical and Biological Engineering, Zhejiang University, Hangzhou, 310027 China; 4 College of Chemical Engineering, Nanjing Tech University, Nanjing, 210009 China; 5 Laboratory for Membrane Materials and Separation Technologies, Shanghai Advanced Research Institute, Chinese Academy of Sciences, Shanghai, 201210 China; 6 Department of Chemical and Environmental Engineering, Yale University, New Haven, CT 06520-8286 USA

**Keywords:** Organic molecules in materials science, Polymers, Synthesis and processing

## Abstract

Microporous organic nanotubes (MONs) hold considerable promise for designing molecular-sieving membranes because of their high microporosity, customizable chemical functionalities, and favorable polymer affinity. Herein, we report the use of MONs derived from covalent organic frameworks to engineer 15-nm-thick microporous membranes via interfacial polymerization (IP). The incorporation of a highly porous and interpenetrated MON layer on the membrane before the IP reaction leads to the formation of polyamide membranes with Turing structure, enhanced microporosity, and reduced thickness. The MON-modified membranes achieve a remarkable water permeability of 41.7 L m^−2^ h^−1^ bar^−1^ and high retention of boron (78.0%) and phosphorus (96.8%) at alkaline conditions (pH 10), surpassing those of reported nanofiltration membranes. Molecular simulations reveal that introducing the MONs not only reduces the amine molecule diffusion toward the organic phase boundary but also increases membrane porosity and the density of water molecules around the membrane pores. This MON-regulated IP strategy provides guidelines for creating high-permeability membranes for precise nanofiltration.

## Abstract (web summary)

Microporous organic nanotubes (MONs) hold considerable promise for designing molecular-sieving membranes because of high microporosity, customizable chemical functionalities, and favorable polymer affinity. Here, the authors report the usage of MONs derived from covalent organic frameworks to engineer 15-nm-thick microporous membranes via interfacial polymerization.

## Introduction

The increasing prevalence of water shortages and contamination severely impedes the sustainable development of industrial and societal activities^1–3^. Rational treatment of non-traditional water sources (e.g., wastewater, seawater, or brackish water) enables abundant freshwater supplies on a global scale^4–6^. However, these complex solutions frequently contain high concentrations of inorganic salts and deleterious trace ions (e.g., boric acid and phosphoric acid). As a result, these solutions are highly difficult to implement properly in a sustainable and low-cost way^7,8^. Because hydrated ions and small waterborne contaminants are typically of sub-nanometer size^9^, there is a need to design microporous membranes that can achieve highly efficient and precise solute separations^10–13^. While commercially established nanofiltration (NF) membranes are commonly used for liquid separations, these membranes offer little control of pore morphology and charge uniformity. These limitations hinder further developments of membranes for high-precision separations of ions and solutes^14^.

Polyamide-based NF membranes are one category of microporous membranes that has been extensively used in liquid-based molecule/ion separations^15^. The polyamide membrane selective layer is constructed through an IP between two reactive monomers which are separately present in two immiscible phases^16^. During the IP process, the diamine-based molecules diffuse from an aqueous phase into an organic phase interface, which contains monomers with acyl chloride groups that initiate the fast and irreversible Schotten-Baumann reaction^17,18^. Notably, the porous support under the formed IP layer causes uneven diffusion of diamine monomers through the non-uniform support pores, leading to the formation of an uncontrolled reaction boundary. This leads to the formation of a rough and thick selective layer with non-uniform pores^19^. Thus, optimizing the support properties to better control the IP process is critical to the fabrication of high-permeability and precise-sieving NF membranes^20,21^.

In this context, incorporating functional nanomaterials such as inorganic nanostrands^22^, metal-organic frameworks (MOFs)^23^, covalent organic frameworks (COFs)^24^, and cellulose nanocrystals^25^ on top of the porous support can enrich the diamine monomers in the IP process via absorptive or adsorptive effects and control the diamine diffusion through the nanomaterial pores^17^. These nanofillers can also block polyamide penetration into the support pores and produce ultrathin and uniform polyamide nanofilms with distinctly improved free-volume elements^23^. These improved features in NF membranes have been shown to enhance solvent permeance without significantly compromising solute selectivity^26^. However, the choice of materials usually neglects pore size matching between the nanofillers and a membrane as well as polymer affinity, which play pivotal roles in solute selectivity and interfacial adhesion between the selective thin-film and underlying support^27,28^.

A recently developed class of porous organic materials, microporous organic nanotubes (MONs), display well-defined structures, high microporosities, tunable chemical functionalities, and excellent hydrothermal stabilities^29,30^. The use of MONs has been broadly extended to catalysis^31^, gas adsorption^32^, and electrochemical devices^33^, among other applications^34^. A recent study reported the controllable formation of organic nanotubes with a narrow pore size distribution, obtained through a selective disassembly of 2D covalent organic frameworks^35^. Constructed with organic components composed of stable covalent bonds, the polymer affinity and stability of MONs derived from COFs are superior to those of other inorganic nanotubes. In light of these features, we surmise that introducing a highly porous and interpenetrated layer of MONs onto a porous support can not only fine-tune the IP process, but also offer additional nanochannels for water transport in the membrane.

**Fig. 1:**
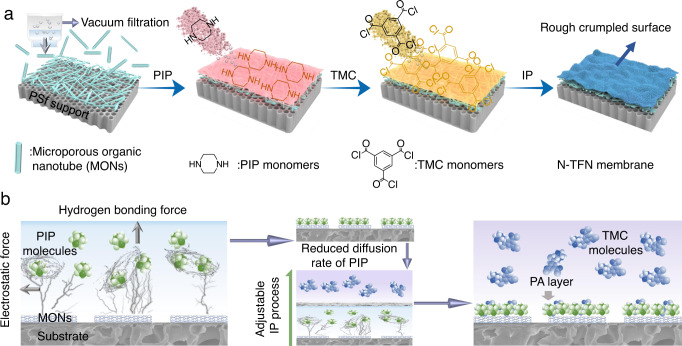
Schematic diagram for the fabricating of MON-regulated thin-film nanocomposite (N-TFN) membrane. **a** preparation process of N-TFN membrane. The organic nanotubes are first loaded onto a polysulfone (PSf) support before piperazine (PIP) and trimesoyl chloride (TMC) are added, triggering the IP process. **b** Schematic depiction of the IP process modulated by hydrogen bonding and electrostatic interactions.

Herein, COF-derived microporous nanotubes were assembled to form a highly porous layer via filtration-assisted assembly, precisely regulating IP to yield MON-based polyamide membranes (Fig. 1a). Selective acid hydrolysis of the boron ring in the dual-pore COF with ethyoxyl groups (COF-OEt) is implemented to generate hydrazone-linked organic nanotubes^35^. In contrast to carbon nanotubes, the use of MONs offers distinct advantages: (i) superior polymer affinity, which prevents the formation of non-selective defects; (ii) pore aperture properties that match those of the formed NF membrane, providing additional selective transport channels; and (iii) structural flexibility, which improves the interfacial adhesion between the selective thin-film and underlying support. Because of the high porosity, large specific surface area, and hydrophilic groups present in the MONs, defect-free, 15-nm-thick polyamide nanofilms with improved microporosity and separation performance are achieved. Notably, the MONs enable enrichment of diamine storage and reduction of diamine diffusion in the IP reaction (see Fig. 1b), as corroborated with molecular simulations.

## Results

### Fabrication of COF-OEt and organic nanotubes

**Fig. 2:**
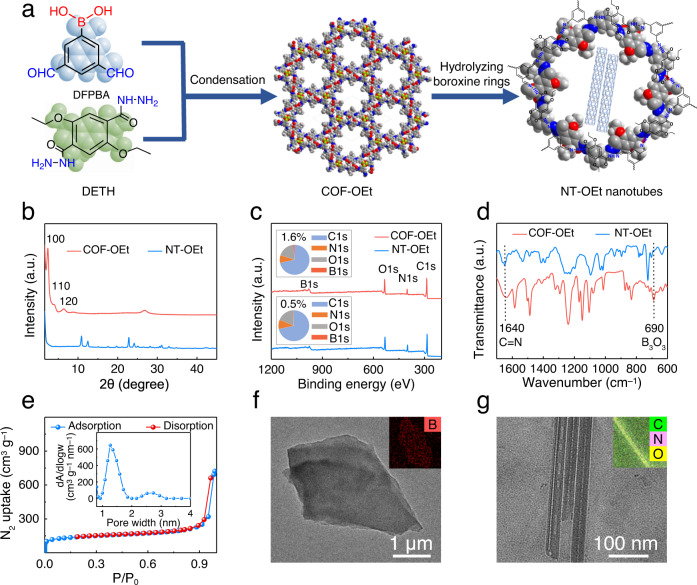
Characterization of COF-OEt and NT-OEt materials. **a** Illustration of the condensation reaction used to produce the materials. **b** PXRD patterns, **c** XPS spectra, and **d** FTIR spectra of COF-OEt and NT-OEt. **e** N_2_ adsorption-desorption isotherm (77 K); inset figure is the PSD profile of NT-OEt. Representative TEM images of **f** COF-OEt and **g** NT-OEt. The insets show elemental analyses obtained via high-resolution transmission electron microscopy (HR-TEM) for the powders.

A 2D COF-bearing ethyoxyl group (COF-OEt) was fabricated by the reaction of 3,5-diformylphenylboronic acid (DFPBA) with 2,5-diethoxyterephthalohydrazide (DETH) (Fig. 2a and Supplementary Figs.1 and 2)^35^. Selective acid hydrolysis of COF-OEt was then used, suspending the COF-OEt in an acidic solution to fabricate the microporous organic nanotubes (termed NT-OEt). Powder X-ray diffraction (PXRD) analyses confirm the successful synthesis of the predicted COF-OEt crystalline structure. The experimental PXRD profile showed a strong diffraction peak at 2.42°, as well as several weak peaks at 4.05°, 6.59°, 8.85°, 11.50° and 22.27°, (Fig. 2b and Supplementary Fig. 3). Figure 2b indicates that the NT-OEt has a comparable crystallinity after the hydrolysis of the B_3_O_3_ rings in COF-OEt.

X-ray photoelectron spectroscopy (XPS) spectra reveal that the COF-OEt was composed of a small amount of boron (1.6%) in addition to larger amounts of carbon (71.7%), nitrogen (8.2%), and oxygen (18.5%) (Fig. 2c and Supplementary Fig. 4). After hydrolysis, the relative boron content decreased in the organic nanotubes (0.5%), according to XPS results. This decline is related to the formed boron oxides upon COF hydrolysis, part of which were then rinsed off. Fourier transform infrared (FTIR) spectra of the COF-OEt and NT-OEt materials are shown in Fig. 2d. The emergence of imine and B_3_O_3_ bands at ~1640 cm^−1^ and ~690 cm^−1^, respectively, reveals the formation of imine bonds and boroxines. In contrast, the peak intensity corresponding to boroxine rings decreased in the NT-OEt FTIR spectrum, whereas the hydrazone peak remained present.

Nitrogen gas adsorption-desorption was utilized to evaluate the porosities of COF-OEt and NT-OEt. The Brunauer-Emmett-Teller (BET) surface area of COF-OEt was 157.4 m^2^/g (Supplementary Fig. 5a). Pore size distribution (PSD) measurement shows that the average pore size around 17.3 Å (Supplementary Fig. 5b). After hydrolysis, due to the formed tubular structure, the surface area of NT-OEt increased to 507.6 m^2^/g. The NT-OEt PSD result demonstrates that the main distributions are primarily at 12.5 Å and 27.5 Å, corresponding to the pore aperture in the walls and hollow structure, respectively (Fig. 2e). As revealed by zeta potential measurements, the nanotube surfaces were more negatively charged (‒10.7 mV) compared to the COF-OEt surfaces (‒1.47 mV), owing to the acid hydrolysis of B_3_O_3_ (Supplementary Fig. 6).

The structures of COF-OEt and NT-OEt were visualized using transmission electron microscopy (TEM). As shown in Fig. 2f and Supplementary Figs. 7 and 8, the COF-OEt displayed a lamellar structure with boron evenly distributed throughout the COF surface (HR-TEM, Fig. 2f inset). After hydrolysis to form the NT-OEt nanotubes, a hollow tubular morphology clearly emerged (Fig. 2g and Supplementary Fig. 9a‒c). The average diameter of the NT-OEt nanotubes was 5.0 ± 0.5 nm, with C, N, and O elements uniformly distributed throughout the NT-OEt (Fig. 2g inset and Supplementary Fig. 10). The uniform elemental distribution and hollow tubular microstructure demonstrate successful formation of the organic nanotubes. Aqueous suspensions of NT-OEt also remain dispersed at room temperature even after 12 hours (Supplementary Fig. 9d).

### Membrane fabrication, structure, and morphology

The COF-derived organic nanotubes were used to synthesize molecular-sieving membranes, by regulating the interfacial polymerization process. Prior to the IP reaction, a layer consisting of the NT-OEt organic nanotubes was first positioned on a polysulfone support via vacuum-assisted filtration (Fig. 1a); the as-prepared membranes are shown in Supplementary Fig. 11. These hydrophilic, highly interpenetrated MONs were selected to optimize the reaction interface, with the goal of forming high-performance polyamide membranes (Fig. 1b).

**Fig. 3:**
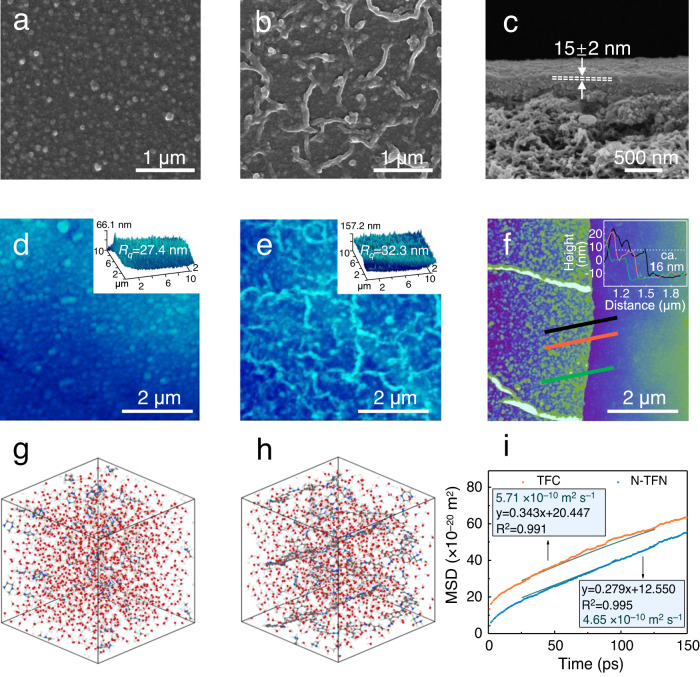
Membrane morphologies altered by the interfacial polymerization process. Control TFC membrane: **a** surface SEM and **d** AFM images (inset: root‒mean‒square roughness, *R*_*q*_). N-TFN membrane: **b** surface SEM image, **c** cross-sectional SEM image, **e** AFM image (inset: root‒mean‒square roughness, *R*_*q*_), and **f** AFM image and height profile (insert) of freestanding N-TFN membranes. The N-TFN membranes shown were prepared using a NT-OEt loading of 2.6 μg cm^−2^. MD simulations of diamine molecules diffusion in water and in the nanotube-water system: **g** simulation box including the PIP monomers in water only, **h** simulation box including the PIP monomers in water with the NT-OEt nanotubes, **i** the MSD curves for the PIP molecules in the two simulation box systems, with the slope of the linear regression line used to calculate PIP diffusivities.

As evident in SEM images (Fig. 3a), conventional IP carried out atop an unmodified polysulfone support leads to thin-film composite (TFC) membranes with small globular structures present on the top surface. Atomic force microscopy (AFM) analyses of the unmodified TFC also show a smooth and nodular surface structure with a relatively low roughness of 27.4 nm (Fig. 3d insert). In comparison, polyamide membranes incorporated with COF-OEt (C-TFN membranes) show a much rougher surface, with larger spots originating from COF-OEt nanoaggregates (Supplementary Fig. 12). As shown in Supplementary Fig. 13, NT-OEt nanotubes were loaded evenly on the supports surface. In-situ IP carried out on an interlayer of the COF-derived nanotubes gives rise to N-TFN membranes with stripe-shaped crumpled surfaces that exhibit fewer globules (Fig. 3b). N-TFN membranes prepared with larger quantities of NT-OEt also exhibited increased amounts of the stripe-shaped structures (Supplementary Figs. 14–15c). AFM analyses further reveal a striped and heterogeneous surface on the N-TFN membranes, with a roughness (32.3 nm) higher than that of the unmodified TFC (Fig. 3e insert).

As recently reported, the generation of stripe-shaped Turing structure is based on diffusion-driven instability, which is typically formed when the diffusion coefficient of the inhibitor molecule (TMC here) is higher than that of the activator molecule (PIP here)^17^. In this work, the NT-OEt layer was employed to impede the PIP diffusion via electrostatic and hydrogen-bonding interactions. Accordingly, this resultant diffusion-driven instability contributes to the formation of the stripe-shaped polyamide surface on the N-TFN membranes.

Cross-sectional SEM images demonstrate that the film thickness reduces from ~118 nm for the TFC membrane to ~ 15 nm upon NT-OEt incorporation (Fig. 3c and Supplementary Fig. 16a‒c). To further probe the thickness and stability of the N-TFN membranes, support-free N-TFN polyamide membrane nanofilms were isolated by immersing the N-TFN membranes in DMF solvent to dissolve the polysulfone support, followed by rinsing with ethanol. The nanofilm transferred to an anodic aluminum oxide support was found intact, thin, and highly transparent (Supplementary Fig. 16d). The nanofilm was also transferred onto a silicon wafer to precisely confirm the film thickness via atomic force microscopy (AFM, Fig. 3f and Supplementary Figs. 17–19). AFM height profiles revealed that the N-TFN nanofilm thickness was indeed ~16 nm (Fig. 3f), while the control TFC polyamide membranes were much thicker (~120 nm, Supplementary Fig. 17). The increase of NT-OEt loading promotes the enrichment of initial PIP molecules within the modified polysulfone supports via electrostatic and H-bonding interactions^36^, as demonstrated by the increased PIP storage (see Section 1.2 of the Supplementary Information and Supplementary Fig. 20). Based on the Freger-Srebnik kinetic model^37^, the thickness of the mature polyamide layer closely relates to the thickness of the initially formed film, which is inversely correlated to the initial monomer concentration at the reaction interface. Thus, the increased initial PIP concentration leads to a decrease in the thickness of the incipient nanofilm induced by the accelerated IP reaction, resulting in the formation of thinner mature PA layers. The presence of an incipient film in turn slows down the growth of the PA film by impeding the penetration of PIP molecules. In this stage, the presence of NT-OEt further retards the PIP diffusion and impedes the longitudinal growth of polyamide, resulting in a thinner mature nanofilm.

Molecular dynamics (MD) simulations were conducted to determine the diffusivities of PIP molecules across the water/hexane interface into the organic phase during IP. These simulations were conducted for both pure water systems (Fig. 3g) and water containing the organic nanotubes (Fig. 3h), to properly elucidate transport effects from the NT-OEt^38^. The PIP diffusion coefficients could be measured based on the slope of the mean-azimuth shift (MSD) curve, using Einstein’s relations (Fig. 3i)^39^. The calculated PIP diffusivity in the N-TFN membrane was 4.65 × 10^−10^ m^2^ s^−1^, 18.6% lower than that in pure water (5.71 × 10^−10^ m^2^ s^−1^). These simulations confirm that the NT-OEt slows the diffusion rate of the diamine molecules during IP.

### Membrane physicochemical properties

**Fig. 4:**
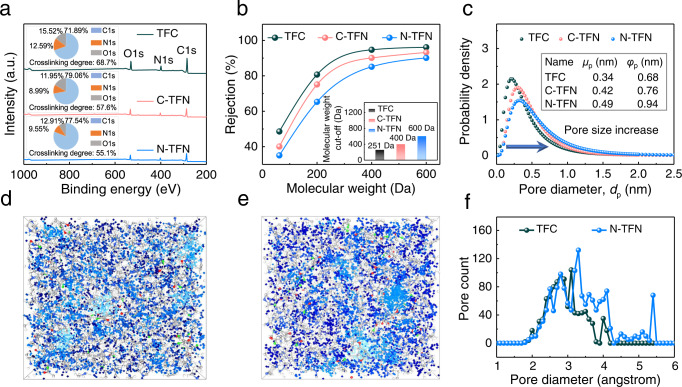
Chemical structures of TFC, C-TFN, and N-TFN. **a** Wide-scan XPS spectra (inset: elemental compositions). **b** Rejection of uncharged model glycol and polyethylene glycol solutes (the feed concentration is 1000 ppm); inset figure is molecular weight cut-off of the membranes. **c** Pore size distributions determined from solute rejection data. Molecular dynamics simulations to elucidate the pore structure and size of TFC and N-TFN membranes: interconnected (dark blue) and disconnected (light blue) voids in **d** TFC and **e** N-TFN polyamide membranes as determined by a probe with a 1.2 Å radius. **f** Simulated pore size distributions of TFC, and N-TFN.

The chemical structures of as-fabricated membranes were measured by ATR-FTIR spectra (Supplementary Fig. 21). Two strong peaks at 1626 cm^−1^ and 3421 cm^−1^ were assigned to C=O stretching from the amide I band and N–H stretching vibration, respectively, revealing the formation of the polyamide. XPS spectra of as-fabricated membranes are presented in Fig. 4a and Supplementary Figs. 22 and 23. According to the obtained O/N ratios, the calculated crosslinking degrees of the TFC, C-TFN, and N-TFN membranes are 68.7%, 57.6%, and 55.1%, respectively (see Section 1.3 of the Supplementary Information). The polyamide surface of N-TNF membranes contains fewer amino groups and more carboxyl groups, which leads to an increased O/N ratio and thus corresponds to a lower crosslinking degree^40,41^. These results reveal that the introduced NT-OEt reduces the crosslinking degree of polyamide films, which enhances water permeability. As discussed previously, the introduced NT-OEt slows down the PIP molecule diffusion toward the organic phase boundary during the diffusion-driven reaction stage, resulting in a polyamide nanofilm with a decreased crosslinking degree.

Deconvolution of the C1s XPS narrow-scan spectra identifies four chemical peaks in each membrane: C=C (284.8 eV), C–C/C–N (286.0 eV), N–C=O (287.9 eV), and O–C=O (288.3 eV) (Supplementary Fig. 23a). This result further confirms the formation of the polyamide layer. Notably, N-TFN membranes exhibited the highest percentage of carboxyl groups (–COOH) of the membranes (Supplementary Table 1), suggesting a high hydrophilicity from these polar groups. The N–H content of the membrane surfaces declined from 20.1% to 16.9% upon incorporation of the NT-OEt nanotubes (Supplementary Fig. 23b and Supplementary Table 2). The surface charge of as-fabricated membranes was also assessed through zeta potential measurements, as surface charge has an important impact on ion separation. Supplementary Fig. 24 shows that N-TFN membranes are more negatively charged than the control TFC and C-TFN membranes, due to the higher amounts of terminal carboxyl groups in the N-TFN membranes. Incorporation of NT-OEt into the NF membranes also led to decreased water contact angles, suggesting concomitant improvements in water transport and antifouling properties (Supplementary Fig. 25). Thermogravimetric analyses indicate that incorporation of COF-OEt and NT-OEt does not decrease the thermal stability of TFN membranes (Supplementary Fig. 26).

To better understand the membrane pore characteristics, molecular weight cut-off (MWCO) analysis was performed using neutral glycol and polyethylene glycol solutes (see Section 1.4 of the Supplementary Information and Fig. 4b). Our results demonstrate that introduction of the NT-OEt layer tunes the MWCO of the membranes from 251 to 600 Da (Fig. 4b inset). This MWCO difference corresponds to an increased average pore diameter from 0.34 to 0.49 nm (Fig. 4c). The larger pore size of the N-TFN membrane is in good agreement with its lower crosslinking degree. The free-volume distributions of polyamide membranes were also analyzed using positron annihilation lifetime spectroscopy (PALS, Supplementary Fig. 27). These results show free-volume enhancements in the N-TFN polyamide films, consistent with those attained by fitting the MWCO data.

Molecular dynamics simulations were also performed to more rigorously confirm the relation between the physicochemical and molecular transport properties of polyamide membranes^42^. A realistic structural model was first constructed to use the Polymatic program. Simulation details are provided in the Supporting Information; the results are displayed in Figs. 4d‒f and Supplementray Fig. 28. Through inserting a theoretical probe of 1.2 Å radius^43^, the connected free-volume regions of the polyamide nanofilms entering to the probes were identified (highlighted in blue in Fig. 4d‒e). The control TFC membranes exhibited a large concentration of isolated voids and thus low inner pore interconnectivity (Fig. 4d). In contrast, the N-TFN membranes showed higher porosity and better pore interconnectivity (Fig. 4e). Pore size distributions obtained from the MD simulations indicate that the TFC and N-TFN membranes have average pore sizes of 0.29 and 0.37 nm, respectively (Fig. 4f and Supplementary Fig. 28), slightly smaller than the experimentally estimated pore diameters based on the solute rejection data. Both simulation and experimental results confirm that the N-TFN membranes possess higher free volume and porosity than traditional TFC membranes.

### Membrane separation performance

**Fig. 5:**
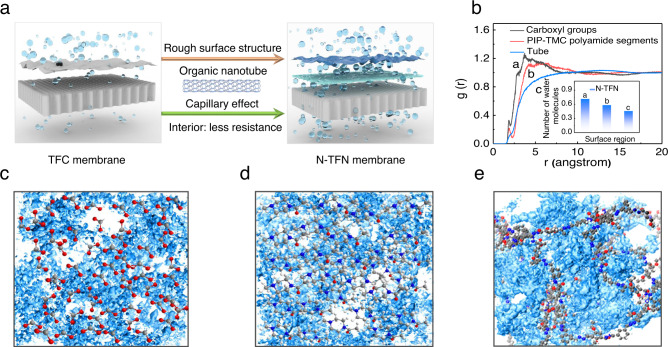
Water transport properties across the N-TFN membrane. **a** Schematic illustration of the increased water absorption and transport through N-TFN membranes. **b** Radial distribution functions for water and three segments in the N-TFN membranes. Insert is the number of water molecule near each segment. Different segments in N-TFN membranes investigated in MD simulations: **c** carboxyl groups (segment a), **d** PIP-TMC polyamide segments (segment b), and **e** tube segments (segment c). The blue background refers to the distribution of water molecules in different regions of the polyamide film.

We performed MD simulations to explore the water transport properties across the polyamide membranes with/without the addition of NT-OEt. The simulations demonstrate the water density distribution and water binding energetics on the membrane surfaces (Fig. 5 and Supplementary Figs. 30–34). Carboxyl groups, PIP-TMC polyamide fragments, and organic nanotube fragments in the N-TFN membrane are expected to serve as the molecular water transport sites, which we denote as segments a, b, and c, respectively^4^. Based on the simulated radial distribution functions, the number of water molecules per functional group near segments a, b, and c were 0.72, 0.58, and 0.46, respectively (Fig. 5b). In the TFC membranes, the water transport pathways were provided by segments a and b, and the simulated number of water molecules around these segments were 0.59 and 0.45, respectively (Supplementary Fig. 30). These results reveal that the introduction of organic nanotubes not only enhances the number of transport sites for water molecules, but also increases the number of water molecules transported across a membrane. Figure 5b shows that the peak values for the number of water molecules in the first layer (*r* = 1.8 Å) in segment a (0.35) is much higher than those in segments b (0.20) and c (0.10). These differences suggest that the water binding capacity in the different segments is as follows: a > b > c (Fig. 5c–e). The magnitudes of the calculated water binding energies obtained from the MD simulations also follow these trends: –134.5 kJ/mol in segment a, –75.3 kJ/mol in segment b, and –70.5 kJ/mol in segment c (see Section 1.6 of the Supplementary Computational simulations). MD simulations were also employed to determine the position of water molecules in a single nanotube. The results showed that about 32% of water molecules passed through the nanotube, further demonstrating the strong capillary effect of nanotubes on water molecules (Supplementary Fig. 34).

**Fig. 6:**
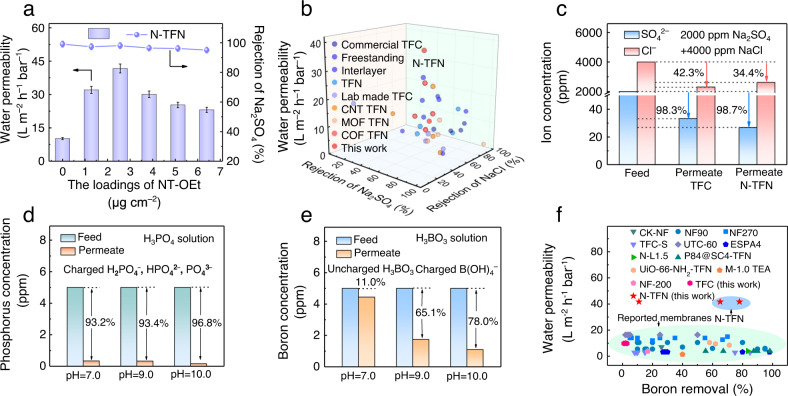
Filtration performance of N-TFN membranes. **a** Water permeability and Na_2_SO_4_ rejection of N-TFN membranes fabricated with different NT-OEt contents (1000 ppm Na_2_SO_4_, 4 bar, pH 7); error bars in the figure represent standard deviation. **b** Performance comparison between N-TFN membranes and advanced polyamide membranes. **c** Ion separation performances of the polyamide membranes for feed solutions containing 2000 ppm Na_2_SO_4_ and 4000 ppm NaCl. **d** Rejection of phosphorus by N-TFN membranes (feed solution: 5 ppm phosphorus). **e** Rejection of boron by N-TFN membranes (feed solution: 5 ppm boron). NaOH was used to adjust the solution pH. **f** Comparison of boron rejection between the N-TFN membranes and state-of-the-art membranes. Unless otherwise stated, all N-TFN membranes were prepared with a NT-OEt content of 2.6 μg cm^−2^, and all tests were measured at a pressure of 4 bar and a temperature of 25 °C.

Guided by the simulation results, we evaluated the filtration performance of the as-fabricated membranes. Each experimental test was conducted after one hour of membrane pre-compaction to ensure steady-state conditions (Supplementary Fig. 35). The impacts of COF-OEt and NT-OEt loading mass on membrane permselectivities were studied using aqueous feed solutions containing 1000 ppm Na_2_SO_4_. The data show that the N-TFN membranes exhibit improved separation performance compared to the C-TFN and traditional TFC polyamide membranes. Increasing the NT-OEt loading mass from 0 to 2.6 μg cm^−2^ resulted in a significant increase in water permeability (10.2 to 41.7 L m^−2^ h^−1^ bar^−1^), while maintaining excellent Na_2_SO_4_ rejection (>98.0%, Fig. 6a). This pronounced increase in water permeability is ascribed to the increased pore size and reduced film thickness of the N-TFN, as well as to the increased water transport pathways provided by the uniformly crumpled surface and porous nanotubes. TEM images for the loading of NT-OEt with different contents on the PSf support are shown in Supplementary Fig. 36. The thickness of the NT-OEt mesh is lower than 65 nm when the loading mass is below 3.8 μg cm^−2^. This indicates that the introduced MONs would not give rise to a distinct mass transfer resistance due to their hollow and porous structure as well as relatively loose distribution. Nonetheless, N-TFN membranes fabricated with higher NT-OEt content showed reduced salt retention, likely due to the formed defects caused by the NT aggregates. Notably, the C-TFN membranes displayed lower water permeances than the N-TFN membranes. For example, the maximum water permeability observed for the C-TFN membranes was only 30.0 L m^−2^ h^−1^ bar^−1^ (Supplementary Fig. 37).

To further explore the ion selectivity of as-fabricated membranes, the water permeability and salt rejection were tested based on different feed solutions containing 1000 ppm of a single salt species: Na_2_SO_4_, MgSO_4_, CaCl_2_, MgCl_2_, or NaCl (Supplementary Fig. 38). The results show that salt retention of each membrane follows the order of Na_2_SO_4_ > MgSO_4_ > MgCl_2_ ~ CaCl_2_ > NaCl. This sequence matches the trends typically observed in PIP-based polyamide films, owing to the synergy of size exclusion and the Donnan effect^44^. However, the N-TFN membranes demonstrated much lower NaCl rejection than the pristine TFC membranes, while maintaining comparably high Na_2_SO_4_ rejection (Supplementary Fig. 38, right). Furthermore, the N-TFN membrane displayed high retention for Na_2_SO_4_ and MgSO_4_ (98.1% and 95.3%, respectively) as well as high water permeabilities of 40.5 and 40.9 L m^−2^ h^−1^ bar^−1^, respectively.

The performance results discussed above demonstrate that the introduction of NT-OEt organic nanotubes significantly improves water permeability without compromising divalent salt rejections. This difference suggests a reduction in the steric hindrance toward water molecules or monovalent ions in the N-TFN membranes due to the increased pore size after introducing the MONs into a polyamide layer. In contrast, the increased surface negative charges endow the N-TFN membranes with the enhanced electrostatic repulsion of sulfate anions, leading to a comparably high Na_2_SO_4_ rejection (Supplementary Fig. 38). Figure 6b and Supplementary Table 4 compare the filtration performance of the N-TFN membrane in this work to reported performances by advanced membranes. The results reveal that our NT-OEt-modified polyamide membranes show a remarkably high water permeance and superior Cl^−^/SO_4_^2−^ selectivity. Notably, our reported N-TFN membranes display better separation performance compared with COF, metal-organic framework, and CNT modified TFN membranes either reported elsewhere and/or made in this work, due to the fact that MONs have superior polymer affinity^45^, matched pore apertures, as well as the positive effect on the IP process (Fig. 6b).

Given that real saline waters consist of a mixture of inorganic salts^36^, a mixed salt solution containing 2000 ppm Na_2_SO_4_ and 4000 ppm NaCl was used to evaluate ion-ion separation ability. Under these conditions, the N-TFN membrane achieved a lower chloride rejection (34.4%) but higher sulfate rejection (98.7%) compared to the TFC membranes (42.3% and 98.3%, Fig. 6c and Supplementary Fig. 39). The Cl^−^/SO_4_^2−^ selectivity of the N-TFN polyamide membranes was accordingly as high as 50. These results show the great promise of NT-OEt-modified membranes for brine refining and salt recovery applications.

Eutrophication of surface waters is a worldwide problem, which is directly linked to the concentration of phosphorus in water. Phosphorus is the limiting indicator, with concentrations higher than 2 ppm causing algal blooms^46^. To address these issues, we studied the use of N-TFN nanofiltration membranes for phosphorus removal. We used a 5 ppm H_3_PO_4_ feed solution to simulate phosphorus-containing water. Since the speciation of phosphorus in water differs across pH values, the impact of pH on phosphorus removal was also evaluated. Dilute sodium hydroxide solution (0.1 M) was used to adjust the solution pH from 7 to 10. At pH 7, phosphoric acid is present as a mixture of H_2_PO_4_^–^ and HPO_4_^2^^–^ (The pKa_1_ of H_3_PO_4_ is 2.12). Similarly, the phosphorus will predominantly be present as HPO_4_^2^^–^ at pH 9 and pH 10 (pKa_2_ = 7.2, pKa_3_ = 12.36). Increased phosphorus removal (up to 96.8%) was attained by the N-TFN membranes with increased solution pH, with the N-TFN membranes achieving higher phosphorus removal at each tested pH than the TFC membranes (Fig. 6d and Supplementary Fig. 40a). Higher phosphorus removal at higher pH is attributable to the increased charge (Donnan) exclusion. Increasing the feed pH from near neutral pH to alkaline pH caused the TFC and N-TFN membranes to be more negatively charged (Supplementary Fig. 24), further enhancing the Donnan effect. Importantly, at every tested pH condition, the N-TFN membrane reduced the phosphorus concentration to levels far below the established standard for safe discharge (less than 2 ppm)^47^.

We also investigated the removal of trace boron using the fabricated N-TFN membranes. Boric acid (H_3_BO_3_) is a trace pollutant widely present in seawater, groundwater, and many other water sources^48^. Low boron removal efficiency by membranes is largely due to the small size and neutral charge of H_3_BO_3_^49^. Analogous to H_3_PO_4_, B(OH)_3_ becomes anionic B(OH)_4_^−^ under weakly basic conditions (pKa_1_ = 9.24, pKa_2_ = 12.74, pKa_3_ = 13.4). Uncharged H_3_BO_3_ molecules at pH 7 conditions, mostly travel across TFC and N-TFN because their pore sizes are much larger than H_3_BO_3_ (Fig. 6e and Supplementary Fig. 40b). After converting the uncharged H_3_BO_3_ molecules to charged B(OH)_4_^−^ anions, the N-TFN membranes showed a distinctly enhanced boron removal performance (78.0% at pH 10), higher than that by TFC membranes (73.2%). Compared to conventional nanofiltration (Fig. 6f and Supplementary Table 5), the N-TFN membranes demonstrate high potential for application for trace boron removal while still maintaining a high water permeability (~40 L m^−2^ h^−1^ bar^−1^).

The long-term stability of N-TFN membranes was also examined via testing water permeability and Na_2_SO_4_ rejection performance over 100 hours (Supplementary Fig. 41a). The N-TFN membranes exhibited exceptional operational stability during the entire 100-hour cross-flow filtration test. At each monitored time, the Na_2_SO_4_ rejection remained above 98%, and water permeability remained between 40.7 and 41.5 L m^−2^ h^−1^ bar^−1^. The water flux of the N-TFN membrane also increased linearly with elevated pressure, demonstrating strong structural stability against hydraulic pressure (Supplementary Fig. 41b). Furthermore, after 20 cycles of performance tests, the permeability of the N-TFN shows a slight decrease to 38.5 L m^−2^ h^−1^ bar^−1^, while maintaining a comparably high Na_2_SO_4_ rejection (Supplementary Fig. 42). This result demonstrates an excellent recovery capability of MON-modified polyamide membranes, which holds potential for water-related industrial applications.

## Discussion

We report the use of MONs (COF-derived organic nanotubes) as an interlayer to regulate interfacial polymerization, enabling the construction of high-performance, molecular-sieving membranes. Introducing the highly porous and interpenetrated layer of NT-OEt nanotubes enables fine-tuning of the IP reaction by enriching the amine monomer storage and slowing down the monomer diffusion rate during IP. These effects lead to 15-nm-thick N-TFN polyamide membranes with striped-shape Turing structures. The N-TFN membranes display higher hydrophilicity, roughness, and negative charge than traditional control polyamide membranes. In addition, the microporous NT-OEt nanotubes enable enhanced pore sizes and free volumes in the N-TFN membranes, as confirmed by both experimental data and MD simulations. These combined features allow the N-TFN membranes to attain exceptional separation performances: for example, high water permeability (up to 41.7 L m^−2^ h^−1^ bar^−1^) and a high Cl^−^/SO_4_^2−^ selectivity for mixed salt solutions. Furthermore, the N-TFN membrane evinces a high removal efficiency for both phosphorus and boron under alkaline conditions, far exceeding the permeances of advanced NF membranes. Our proposed process for regulating IP using MONs provides a useful tool for the design of polyamide membranes for precise nanofiltration.

## Methods

### Materials and chemical reagents

Materials and chemical reagents can be found in Supplementary Information (Supplementary Experimental Methods 1.1).

### Synthesis of COF-OEt and organic nanotubes

3,5-diformylphenylboronic acid (DFPBA, 10.0 mg, 0.056 mmol) and 2,5-diethoxyterephthalohydrazide (DETH, 16.2 mg, 0.056 mmol) monomers were synthesized in a glass beaker with a mixture of n-butanol and o-dichlorobenzene (1/1, 1.5 mL). First, the glass beaker was sealed under vacuum after a freeze-pump-thaw cycle. The beaker was then heated at 150 °C for 3 days to obtain yellow colloids. After cooling to room temperature, the colloids were collected and dried at 120 °C in a vacuum drying oven for 4 hours, yielding the COF crude product. This synthesized COF was further washed with ethanol and methanol (1/1, 8 mL) before drying to obtain yellow COF-OEt microcrystals. Finally, the COF was added to an aqueous HCl solution (6 M), which was then set aside at least one week. The solids were filtered, washed with ethanol, and dried to obtain the NT-OEt organic nanotubes.

### Fabrication of TFC and N-TFN membranes

TFN membranes were prepared using PSf membranes as porous supports. At 0.2 bar, 10 mL aqueous suspensions that contain COF-OEt or NT-OEt with different contents were loaded onto a PSf membrane support via vacuum filtration. Then, an IP reaction was subsequently conducted. In detail, at 0.2 bar, a 10 mL 0.1 wt% PIP solution was pumped onto the PSf support. The membrane was then set to dry in air until no water was noticeably present on the PSf support surface. Afterward, a 10 mL 0.1 wt% TMC-n-hexane solution was poured onto the membrane surface to trigger interfacial polymerization. The membrane was left undisturbed for 1 min (25 °C, and the relative humidity was about 60%). The remaining solution was removed from the membrane, and the unreacted TMC solution was removed by using the solvent n-hexane. Finally, the polyamide membrane was stand at 80 °C for 3 min and stored in deionized water before use. TFN membranes prepared with COF-OEt were labeled as C-TFN, while TFN membranes prepared with NT-OEt were labeled as N-TFN. The fabrication route for a TFC membrane is the same as that of a TFN membrane, but without adding nanoparticles.

### Membrane performance tests

A crossflow nanofiltration device with a membrane area of 7.1 cm^2^ was employed to evaluate membrane separation performance. The pure water permeability of each membrane was tested at 4 bar. The flux (*J*, L m^‒2^ h^–1^) of the as-prepared membrane was calculated using

1 $$J=\frac{V}{A\,\triangle t}$$

where *V* (L) is the permeate volume, Δ*t* (h) is the filtration time, and *A* (m^2^) is the effective area of each membrane. The permeability (*P*, L m^‒2^ h^–1^ bar^–1^), defined as the flux per unit applied pressure, was calculated from

2 $$P=\frac{J}{\triangle P}$$

where Δ*P* (bar) is the transmembrane pressure.

To measure the solute rejection of the membranes, the feed solutions were pumped into the crossflow device (4 bar). The solute rejection (*R*, %) and the Cl^–^/SO_4_^2–^selectivity (*α*) were calculated by the following equations:

3 $$R=\left(1-\frac{{C}_{{{{{{\rm{p}}}}}}}}{{C}_{{{{{{\rm{f}}}}}}}}\right) \times 100\%$$

4 $$\alpha=\frac{{P}_{{{\mbox{NaCl}}}}}{{P}_{{{{\mbox{Na}}}}_{2}{{{\mbox{SO}}}}_{4}}}=\frac{1-{R}_{{{\mbox{NaCl}}}}}{{1-R}_{{{{\mbox{Na}}}}_{2}{{{\mbox{SO}}}}_{4}}}$$

where *C*_p_ and *C*_f_ (g L^‒1^) are the salt concentrations of the permeate and feed, respectively, and ${P}_{{{{\mbox{Na}}}}_{2}{{{\mbox{SO}}}}_{4}}$ and *P*_NaCl_ are the permeabilities of Na_2_SO_4_ and NaCl, respectively.

Salt concentration was measured with a conductivity analyzer (Leichi, DDS-11A, China). Ionic concentrations of mixed salt solutions were determined by ion chromatography (Thermo Fisher, USA). Phosphorus and boron concentrations were determined by inductively coupled plasma (ICP-OES, ICPE-9820, Japan).

### Characterization

Details of the various characterization methods can be found in Supplementary Information (Supplementary Experimental Methods 1.5).

### Computational simulations

Detailed computational simulations can be found in Supplementary Information (Supplementary Experimental Methods 1.6).

## Supplementary information

Supplementary Information

## Data Availability

The authors declare that all data supporting the findings of this study are available within the paper and its Supplementary information files or available from the corresponding author upon request.
