# Supplementary material for: Microporous organic nanotube assisted design of high performance nanofiltration membranes
Source: Nat Commun. 2022 Dec 27;13:7954. doi: 10.1038/s41467-022-35681-9 (PMC9794819; doi:10.1038/s41467-022-35681-9)
Supplement: Supplementary file 1 — Supplementary Information [file 41467_2022_35681_MOESM1_ESM.pdf]

Supplementary Information

Microporous Organic Nanotube Assisted Design of High Performance Nanofiltration Membranes

Shuangqiao Han[1], Junyong Zhu[1]*, Adam A. Uliana[2], Dongyang Li[1], Yatao Zhang[1]*, Lin Zhang[3], Yong Wang[4], Tao He[5], Menachem Elimelech[6]*

[1] School of Chemical Engineering, Zhengzhou University, Zhengzhou 450001, China

[2] Department of Chemical and Biomolecular Engineering, University of California, Berkeley, CA 94720, USA

[3] Key Laboratory of Biomass Chemical Engineering, College of Chemical and Biological Engineering, Zhejiang University, Hangzhou 310027, China

[4] College of Chemical Engineering, Nanjing Tech University, Nanjing 210009, P. R. China

[5] Laboratory for Membrane Materials and Separation Technologies, Shanghai Advanced Research Institute, Chinese Academy of Sciences, Shanghai, 201210, China

[6] Department of Chemical and Environmental Engineering, Yale University, New Haven, Connecticut 06520-8286, United States

***Correspondence to:** zhujunyong@zzu.edu.cn;
zhangyatao@zzu.edu.cn;
menachem.elimelech@yale.edu

Table of Contents

1. Supplementary Experimental Methods........5
1.1 Supplementary Materials........5
1.2 Monomer storage capacity measurements of supports........5
1.3 Calculation of membrane surface crosslinking degree........6
1.4 Molecular weight cut-off (MWCO) and pore size distribution........6
1.5 Material characterization methods........7
1.6 Computational simulations........9
1.6.1 Simulated powder X-ray diffraction (PXRD) patterns........9
1.6.2 Molecular dynamics (MD) and mean square displacement (MSD) computations........9
1.6.3 Hole distribution and characterization........10
1.6.4 Radial distribution functions (RDFs) and number of water molecules........11
1.6.5 Water distribution surrounding different segments........11
1.6.6 Water molecules passed through the nanotube........12
2. Supplementary Figures........13
Supplementary Fig. 1 Model images of: (a) COF-OEt and (b) NT-OEt, constructed using Materials Studio........13
Supplementary Fig. 2 Synthesis and structures of COF-OEt and NT-OEt........14
Supplementary Fig. 3 PXRD curves of COF-OEt with different degree: (a) 2–25° and (b) 7–25°........15
Supplementary Fig. 4 The content of C1s, N1s, O1s and B1s of (a) COF-OEt and (b) NT-OEt........16
Supplementary Fig. 5 (a) N_2 adsorption-desorption isotherm of COF-OEt at 77 K. (b) pore size distribution profile of COF-OEt........17
Supplementary Fig. 6 Zeta potentials of COF-OEt and NT-OEt........18
Supplementary Fig. 7 TEM images of COF-OEt with different magnifications. Scale bars: (a) 500 nm (inset image is an aqueous suspension containing COF-OEt with the Tyndall effect), (b) 200 nm........19
Supplementary Fig. 8 HR-TEM of COF-OEt. Scale bar: 1 μm........20
Supplementary Fig. 9 TEM images of NT-OEt. Scale bars: (a) 500 nm, (b) 200 nm, (c) 100 nm. (d) TEM image (scale bar: 100 nm) of NT-OEt at room temperature stored for 12 h (inset image is an aqueous suspension containing NT-OEt with the Tyndall effect)........21
Supplementary Fig. 10 HR-TEM of a single NT-OEt. Scale bars: 100 nm........22
Supplementary Fig. 11 Photo images of the TFC, C-TFN and N-TFN membrane........23
Supplementary Fig. 12 SEM images of the C-TFN membrane top surfaces, prepared with different nanoparticle loadings: (a) 1.3 μg cm^{-2}, (b) 2.6 μg cm^{-2}, (c) 3.8 μg cm^{-2}. Scale bars: 2 μm........24
Supplementary Fig. 13 SEM images of the NT-OEt deposited membrane, prepared by loading 2.6 μg cm^{-2} of NT-OEt onto the PSf support. Scale bars: (a) 1μm, (b) 200 nm........25
Supplementary Fig. 14 SEM images of the N-TFN membrane top surfaces, prepared with different nanoparticle loadings: (a) 0.75 μg cm^{-2}, (b) 1.3 μg cm^{-2}, (c) 3.8 μg cm^{-2}. Scale bars: 2 μm........26
Supplementary Fig. 15 AFM images of (a) TFC, (b) C-TFN, and (c) N-TFN membranes........27
Supplementary Fig. 16 Cross-sectional SEM images of (a) TFC, (b) C-TFN, and (c) N-TFN membranes. (d) A free-standing N-TFN membrane on an anodic aluminum oxide support. Scale bars: 500 nm. ...28
Supplementary Fig. 17 AFM images of freestanding TFC membrane: (a) height image, (b) phase image,

(c) corresponding height profile, (d) 3D image. 29
Supplementary Fig. 18 AFM images of freestanding C-TFN membrane: (a) height image, (b) phase image, (c) corresponding height profile, (d) 3D image...30
Supplementary Fig. 19 AFM images of freestanding N-TFN membrane: (a) height image, (b) phase image, (c) corresponding height profile, (d) 3D image...31
Supplementary Fig. 20 PIP storage capacity of the fabricated membranes. 32
Supplementary Fig. 21 FTIR spectra of the fabricated membranes...33
Supplementary Fig. 22 The content of C1s, N1s, O1s and degree of crosslinking of (a) TFC membrane, (b) C-TFN membrane and (c) N-TFN membrane..34
Supplementary Fig. 23 (a) C1s and (b) N1s XPS spectra of the fabricated membranes.......................35
Supplementary Fig. 24 Zeta potentials as a function of pH of the fabricated membranes....................36
Supplementary Fig. 25 Water contact angles of as-prepared membranes..37
Supplementary Fig. 26 TGA decomposition curves of the fabricated membranes..............................38
Supplementary Fig. 27 Positron annihilation patterns for the TFC and N-TFN membranes................39
Supplementary Fig. 28 The number of rays calculated using the Zeo++ software, for the TFC and N-TFN membranes after interfacial polymerization was performed (see Section 1.6.3 of the Supporting Information for details)..40
Supplementary Fig. 29 (a) SEM image and (b) pore size distribution of PSf support..........................41
Supplementary Fig. 30 (a) Radial distribution functions between water and different segments in the TFC membrane. (b) Numbers of water molecules around the carboxyl groups (segment a) and PIP-TMC polyamide segments (segment b) in the TFC membranes, calculated from MD simulations.....42
Supplementary Fig. 31 Different segments in the TFC membranes revealed by MD simulations in 100 frames. Carboxyl groups: (a) a larger version and (b) a zoomed–in version. PIP-TMC polyamide segments: (c) a larger version and (d) a zoomed–in version. The blue background refers to the distribution of water molecules in different regions of a polyamide film..43
Supplementary Fig. 32 A larger version of different segments in the N-TFC membranes in 100 frames: (a) carboxyl groups, (b) PIP-TMC polyamide segments...44
Supplementary Fig. 33 Different segments in the N-TFN membranes in 500 frames. Carboxyl groups: (a) a larger version and (b) a zoomed-in version. PIP-TMC polyamide segments: (c) a larger version and (d) a zoomed–in version...45
Supplementary Fig. 34 Process of water molecules passing through a nanotube: (a) front and (b) side. 46
Supplementary Fig. 35 Schematic depiction of the N-TFN separation process. 47
Supplementary Fig. 36 TEM images of the MONs mesh deposited in the PSf support for the different loadings (a) 1.3μg/cm^{-2}, (b) 2.6μg/cm^{-2}, (a) 3.8μg/cm^{-2}. 48
Supplementary Fig. 37 Water permeability and Na_2SO_4 rejection of the polyamide membranes fabricated with different COF-OEt contents (feed: 1000 ppm Na_2SO_4, 4 bar, pH = 7). All the error bars in this figure represent the standard deviation of the experiments...49
Supplementary Fig. 38 Water permeability and salt rejection of the pristine TFC membrane and N-TFN membrane for various aqueous solutions containing 1000 ppm of one inorganic salt type. All the error bars in this figure represent the standard deviation of the experiments. 50
Supplementary Fig. 39 Ion chromatograph distribution curves of mixed salts....................................51
Supplementary Fig. 40 (a) Rejection of phosphorus by the TFC membranes (feed solution: 5 ppm phosphorus). (b) Rejection of boron by the TFC membranes (feed solution: 5 ppm boron). The pH was

altered using a 0.1 M NaOH solution. 52
Supplementary Fig. 41 (a) Long-term stability performance of N-TFN membranes (feed: 1000 ppm Na_2SO_4). (b) Water flux and Na_2SO_4 rejection performance by N-TFN at varied pressures. All the error bars in these figures represent the standard deviation of the experiments. 53
Supplementary Fig. 42 Recovery ability of the membrane of N-TFN membranes (feed: 1000 ppm Na_2SO_4 or NaCl solution, 4 bar). All the error bars in this figure represent the standard deviation of the experiments. 54
3. Supplementary Tables 55
Supplementary Table 1 Chemical species compositions of the membrane top surfaces, obtained from C1s XPS spectra..55
Supplementary Table 2 Chemical species compositions of the membrane top surfaces, obtained from N1s XPS spectra. 56
Supplementary Table 3 Properties of the PSf Membrane Support...57
Supplementary Table 4 Comparison of nanofiltration performances between the N-TFN membranes and state-of-the-art polyamide membranes. 58
Supplementary Table 5 Summarized boron removal performance of state-of-the-art NF membrane desalination technology...61
Supplementary Table 6 Summarized phosphorus removal performance of state-of-the-art NF membrane desalination technology...65
Supplementary References..66

1. Supplementary Experimental Methods

1.1 Supplementary Materials

Trimesoyl chloride (TMC > 98.0%), piperazine anhydrous (PIP > 98.0%) and poly(ethylene oxide) (PEO) with different molecular weights were purchased from Adamas reagent co., Ltd., Sodium chloride (NaCl > 99.0%), sodium sulfate (Na_2SO_4 > 99.0%), magnesium sulfate ($MgSO_4$ > 99.0%), magnesium chloride ($MgCl_2$ > 99.0%), and calcium chloride ($CaCl_2$ > 99.0%) were obtained from Aladdin industrial corporation (Shanghai, China). Hydrogen chloride (HCl > 36.0%), *n*-hexane ($C_{10}H_{22}$ > 99.0%) were purchased from Sinopharm Chemical Reagent (Shanghai, China). Ethylene glycol (62.1 Da) and a series of polyethylene glycol (PEG) with different molecular weights were purchased from Kermal, Tianjin, China.

3,5-diformylphenylboronic acid (DFPBA, 99.0%) and 2,5-diethoxyterephthalohydrazide (DETH, 99.0%) were purchased from Shanghai Tengqian Biological Technology Co., Ltd. (Shanghai, China). Polysulfone (PSf) support was provided by Beijing Sapruite Equipment Co., Ltd. (Beijing, China). It is noted that the molecular weight cut-off (MWCO) of PSf membrane support was measured to be 327 KDa, with an average pore diameter of 20.58 nm (Supplementary Fig. 29 and Table 3). Details for measuring the MWCO and pore size of PSf support are shown later in Supplementary Information.

1.2 Monomer storage capacity measurements of supports

A 10 mL 0.1 wt% PIP-water aqueous solution was pumped onto the PSf support at 0.2 bar via vacuum filtration. Then, 1.5 mL of the filtrate solution was extracted at different times, the PIP concentration was measured with a UV-VIS spectrophotometer (UV-1800CHI240V, Japan),

and then reversed.

1.3 Calculation of membrane surface crosslinking degree

To calculate the cross-linking degree of polyamide active layer, the O/N ratio of active layer measured by X-ray photoelectron spectroscopy (XPS) was introduced into the Supplementary Eq. equation (1) [1–2]:

$$\frac{\mathrm{O}}{\mathrm{N}} = \frac{3X + 4Y}{3X + 2Y} \tag{1}$$

respectively. The cross-linking degree (C, %) was then calculated according to Supplementary Eq. equation (2):

$$C = \frac{X}{X + Y} \tag{2}$$

1.4 Molecular weight cut-off (MWCO) and pore size distribution

The MWCO of the PSf membrane support was determined by filtration of uncharged model glycol, polyethylene glycol (PEG, and poly(ethylene oxide) (PEO) with average molecular weights of 62.1, 200, 400, 600, 800, 1000, 20000, 100000, 300000 and 400000 Da, respectively. For PSf support, the rejection (*RE*) of the 200 mg/L solution was evaluated at a pressure of 4 bar. For as-prepared membranes, the *RE* of 1000 mg/L solution was evaluated at a pressure of 4 bar. Uncharged model glycol, PEG, and PEO concentrations were measured using total organic carbon (TOC). The *RE* (%) is calculated from measured feed (C_1, g L^{-1}) and permeate concentration (C_2, g L^{-1}) using the following Supplementary Eq. equation (3):

$$RE = (1 - \frac{C_2}{C_1}) \times 100\% \tag{3}$$

When *RE*=90%, the MWCO of the composite membrane is the molecular weight of PEG

and PEO solute. The pore size information (i.e. pore radius distribution and mean pore radius) was obtained by using the lognormal model between solute retention rate and solute Stokes radius. From the average molecular weight of PEG solute, the Stokes radius can be calculated by the Supplementary Eq. (4–5)[3]:

$$r = 16.73 \times 10^{-12} \times M_{\mathrm{w}}{}^{0.557} \tag{4}$$

For PEO:

$$r = 10.44 \times 10^{-12} \times M_{\mathrm{w}}{}^{0.587} \tag{5}$$

In the equations, M_{W} is in grams g/mol. Assuming that there are no spatial and hydrodynamic interactions between PEG or PEO solute and membrane pores, the pore size distribution of the membrane can be obtained by the following Supplementary Eq. equation (6):

$$\frac{\mathrm{d}R(d_{\mathrm{p}})}{\mathrm{d}d_{\mathrm{p}}} = \frac{1}{d_{\mathrm{p}} \ln \sigma_{\mathrm{p}} \sqrt{2\pi}} \exp[-\frac{(\ln d_{\mathrm{p}} - \ln \mu_{\mathrm{p}})^2}{2(\ln \sigma_{\mathrm{p}})^2}] \tag{6}$$

where μ_{p} is the mean effective pore radius which is determined at the PEG and PEO rejection of RE = 50% and σ_{p} is the geometric standard deviation, which is defined as the ratio of d_{p} at RE = 84.13% over that at RE = 50%. The membrane pore size distribution can then be calculated from the probability density function based on the value of μ_{p} and σ_{p}[4].

1.5 Material characterization methods

Surface physicochemical properties (e.g., elemental composition, thermostability, hydrophilicity, structure and surface charge) and morphology were determined by X-ray photoelectron spectroscopy (XPS), powder X-ray diffractometry (PXRD), atomic force microscopy (AFM), scanning electron microscopy (SEM), Transmission electron microscopy (TEM), thermogravimetric analysis (TGA), attenuated total reflection Fourier transform

infrared (ATR-FTIR) spectroscopy, water contact angle (WCA) goniometry, Brunauer–Emmett–Teller (BET) and zeta potential measurements. PXRD analyses of the membranes were carried out on a PANalytical X′Pert Pro (PANalytical, The Netherlands) in the scanning range of 2θ between 5° and 60° (for materials, the scanning range of 2θ between 0.5° and 30°), using copper K-α as the source of radiation and a step size of 0.02°. XPS (K-alpha, Thermo Fisher, USA) was used to analyze the surface chemical functionality. The cross-sectional and surface morphologies of the membrane were examined with scanning electron microscopy (SEM, JSM-6700F, JEOL, Japan) operating at 10.0 kV. The TGA measurements (NETZSCH TG 209, Germany) were carried out under a nitrogen atmosphere from room temperature to 650 °C, with a heating rate of 10 °C/min. Transmission electron microscopy (TEM, FEI TalosF200S, Czech) was used to examine morphology of samples and the thickness of the MONs mesh deposited for the different loadings. The membrane surface morphologies and roughness parameters were investigated using AFM (Bruker Dimension Fastscan, USA) at the ScanAsyst mode. The WCAs (OCA25, Dataphysics instruments, Germany) of the membranes were measured by a contact angle goniometer (Maist Drop Meter A-100P) equipped with a highspeed charge-coupled device (CCD) camera via the sessile drop method. The zeta potential values of the membranes were recorded through a SurPASS™ 3 electrokinetic analyzer (Anton Paar, Graz, Austria) with an adjustable gap cell. The zeta potential values were determined in a background electrolyte of 1 mM KCl solution, over a pH range from 3 to 10 at room temperature. ATR-FTIR spectroscopy (4000–400 cm^{-1}) was collected on the samples using a FTIR spectrometer (FT-IR, Thermo Nicolet Corporation, USA). The phosphorus and boron concentration were determined by inductively coupled plasma (ICP-OES, ICPE-9820, Japan).

The porosity of the materials was measured by BET (Quantachrome, ASAP 2460 3.01, USA). PIP concentration was measured with a UV-VIS spectrophotometer (UV-1800CHI240V, Japan). The concentration of PEG and PEO solution was determined using a TOC-VCPH analyzer (Shimadzu, Japan) to calculate molecular weight cut-off (MWCO). The free volumes of the TFC and N-TFN membranes were measured by positron annihilation technique (PALS, DPLS3000). The radioactive source is a 22Na isotope with positron energies ranging from 0 to 545 keV.

1.6 Computational simulations

1.6.1 Simulated powder X-ray diffraction (PXRD) patterns

The representative COF-OEt structure, consisting of 6 B, 24 O, 126 C, 24 N, and 114 H atoms, was constructed in a rhombic cell with lattice parameters of a = b = 38.55 Å and c = 4.07 Å. The initial COF-OEt was fully optimized using the Forcite module of Materials Studio. The NT-OEt structure was obtained based on the optimized COF-OEt structure, after completing the following two steps. The B–O six-membered rings of the relaxed COF-OEt optimized structure were deleted, and the unsaturated C atom from the benzene ring was terminated by a H atom. The corresponding simulated PXRD patterns for the COF-OEt structures were computed via the Reflex module of Materials Studio.

1.6.2 Molecular dynamics (MD) and mean square displacement (MSD) computations

Molecular dynamics (MD) simulations were carried out to calculate the diffusion rate of PIP molecules in water solution in the TFC and N-TFN membranes (Fig. 3g and h). The diffusion

coefficients of the PIP molecules in water solution can be estimated from the slope of the mean square displacement (MSD) curves in Fig. 3i, using the Einstein relationship[5]. All MD simulations were performed using the Forcite module in Materials Studio. With a constant PIP: water weight ratio of 15:85, the PIP and water molecules were packed in a cubic simulation box with side length of 40 Å. In a separate analogous simulation, the same PIP/water-containing cubic simulation box was constructed, but six NT-OEt nanotubes were also added. The Forcite module and COMPASS force field were employed to optimize the two above systems, and the MD simulations were run in the NVT ensemble (T = 298 K) with a time step of 1.0 fs and a total simulation time of 300 ps. The data were collected from the final 150 ps for analyzing. Periodic boundary conditions were applied in all three dimensions.

1.6.3 Hole distribution and characterization

PIP and TMC molecules with a weight ratio of 15:85 were packed in one cubic cell with a side length of 40 Å; this system was marked as TFC membrane. For the PIP/TMC/nanotube system (marked as N-TFN membrane), the weight ratio between PIP and TMC was still kept at 15:85. Energy minimization was performed for the above two systems, using the Forcite module of Materials Studio with the COMPASS force field. After obtaining equilibrium structures, Zeo++ [6–8] was used to analyze voids, including pore size distribution with a probe radius of 1.2 Å and pore landscape analysis. Here, the pore landscape was characterized based on the pore count under different pore size and number of rays, which was relative to the probe-accessible space. The pore size distribution data obtained from the Zeo++ code was visualized by the Vislt tool [9].

1.6.4 Radial distribution functions (RDFs) and number of water molecules

PIP, TMC, and water molecules with a molar ratio of 1:1:12 were packed in one cubic cell where each dimension was 40 Å. For the PIP/TMC/water/tube system, the molar ratio of PIP, TMC, and water molecules was still kept at 1:1:12. A general size of 40·40·40 Å was designed to model the membrane consisting of the water/nanotube system, where 8 microporous organic nanotubes were inserted, ensuring the simulation accuracy and computing efficiency. Energy minimization using the Forcite module of Materials Studio with the COMPASS force field was performed for the above two systems. After completing the geometry optimization process for each system, the NVT ensemble (temperature: 298 K) was applied in the MD calculations, with a time step of 1.0 fs and a total simulation time of 100 ps. Frames and snapshots containing the geometry information were selected every 100 steps. From the MD simulations, radial distribution functions (RDFs) of water molecules with different segments were extracted. The number of water molecules surrounding different segments was then calculated based on the RDFs.

1.6.5 Water distribution surrounding different segments

In order to represent water distribution surrounding different segments, all-atom MD simulations were carried out for the PIP/TMC/water and PIP/TMC/tube/water systems previously described. These simulations were performed using the GROMACS software package with the OPLS-AA force field[10,11]. Energy minimization was performed with termination gradients of 10 kJ mol^{-1} nm^{-1}. The equilibrium MD simulation was run for 40 ns

under the NVT ensemble, with a temperature of 298 K and a time step of 1 fs. The periodic boundary conditions were imposed on all three dimensions. From the MD simulations, spatial distribution functions (SDFs) of water molecules were obtained. The SDF data were then visualized using the Visual Molecular Dynamics program.

1.6.6 Water molecules passed through the nanotube

1 ns NVT MD simulation is ran with 100 snapshots–each per 10 ps collected to do the post analysis. A distance of 1.8 Å away from the cross section of the nanotube is applied to detect the water molecules passing through the nanotube. Note that 1.8 Å is a typical length for searching for hydrogen bond, which describes a strong pairwise interactions and needs to be considered in this study. After average of 100 snapshots for all nanotubes and division by the total number of water molecules in the system, a number of 32±2.1% is concluded. Supplementary Fig. 34 shows one typical snapshot of NT-OEts nanotube with water molecules passing through, where Supplementary Fig. 34b also provides a side view of water molecules outside but still within 1.8 Å distance to the nanotube.

2. Supplementary Figures

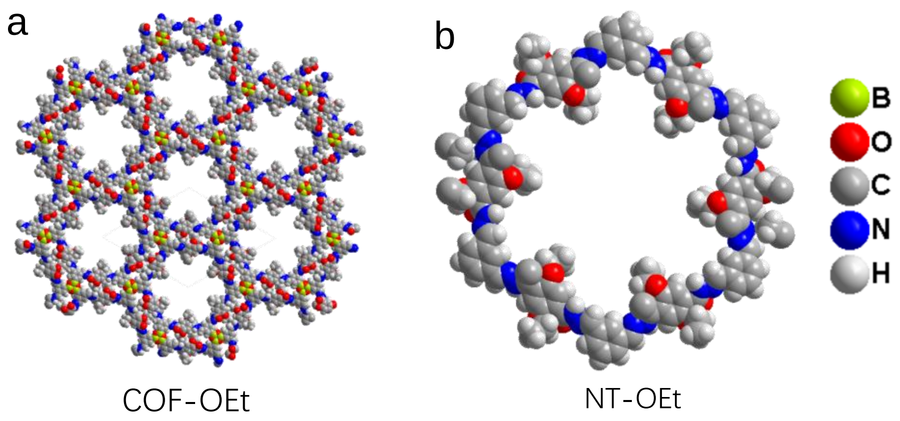

Supplementary Fig. 1 Model images of: (a) COF-OEt and (b) NT-OEt, constructed using Materials Studio.

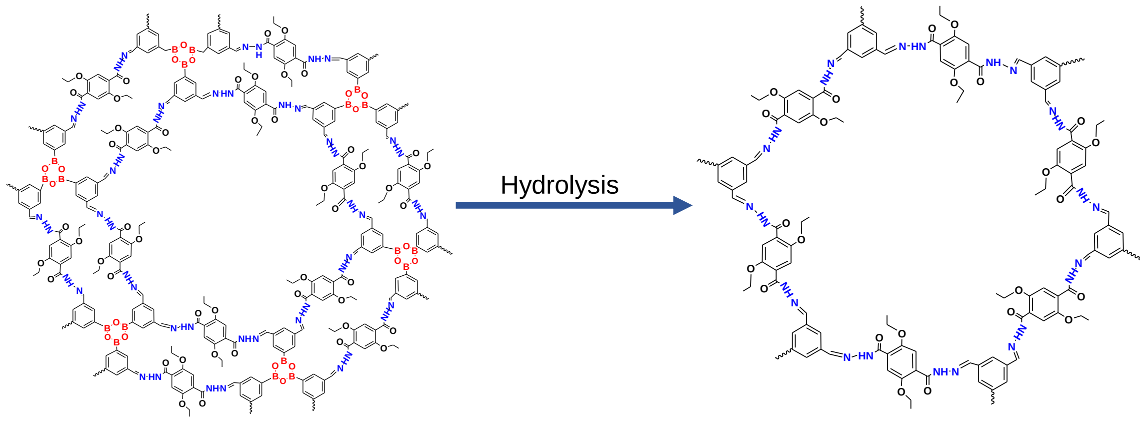

Supplementary Fig. 2 Synthesis and structures of COF-OEt and NT-OEt.

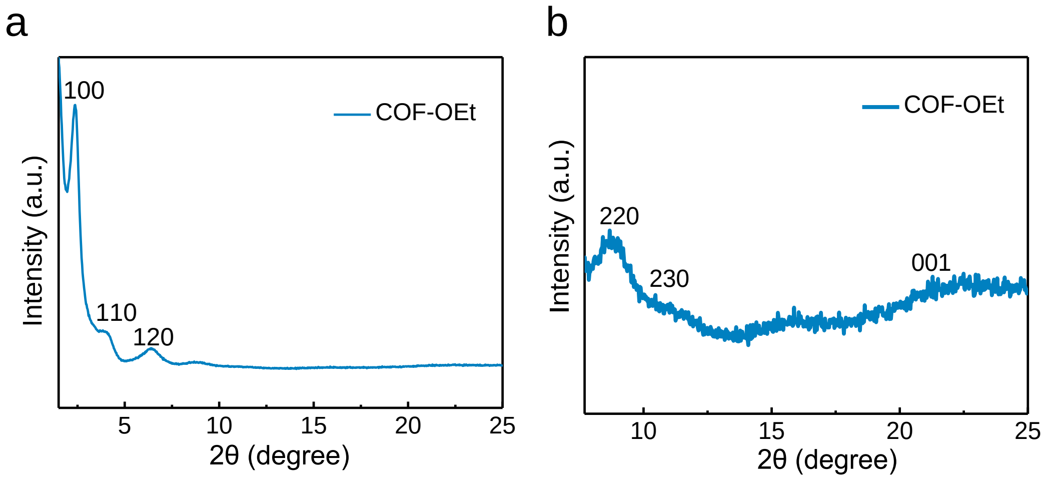

Supplementary Fig. 3 PXRD curves of COF-OEt with different degree: (a) 2–25° and (b) 7–25°.

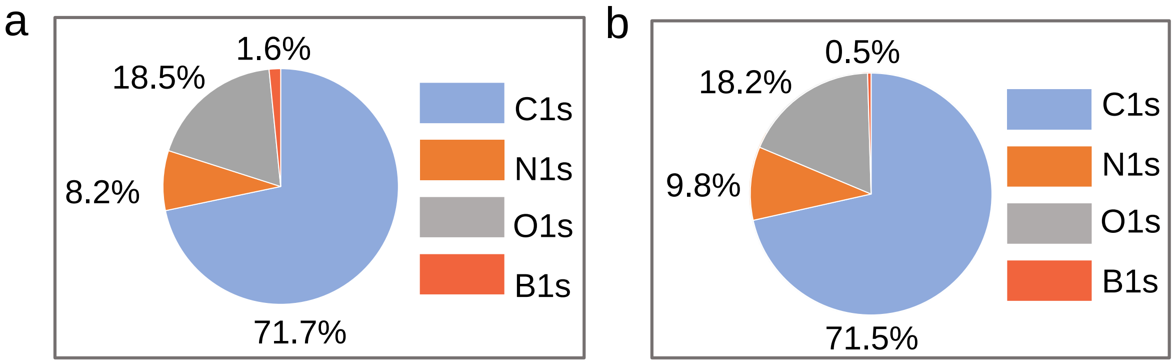

Supplementary Fig. 4 The content of C1s, N1s, O1s and B1s of (a) COF-OEt and (b) NT-OEt.

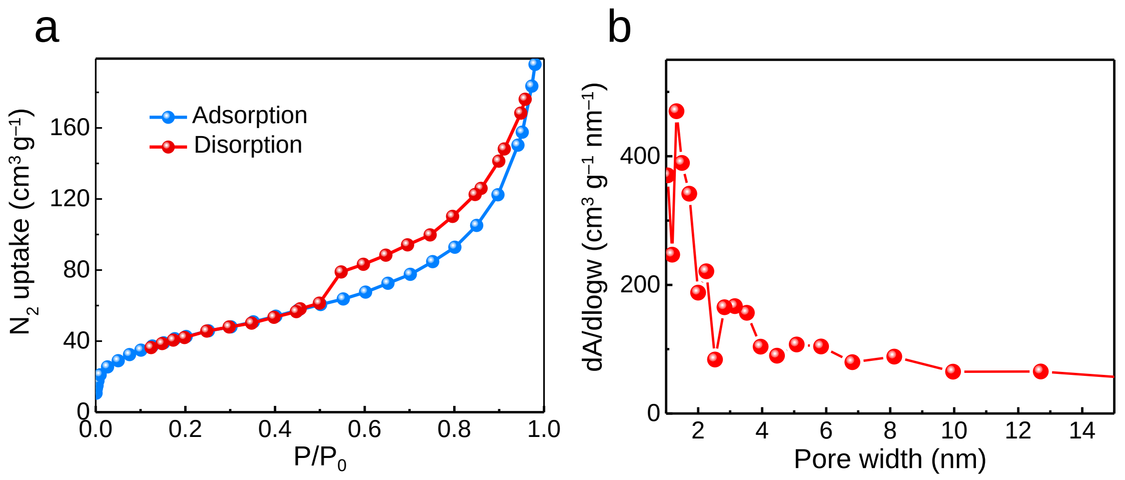

Supplementary Fig. 5 (a) N_2 adsorption-desorption isotherm of COF-OEt at 77 K. (b) pore size distribution profile of COF-OEt.

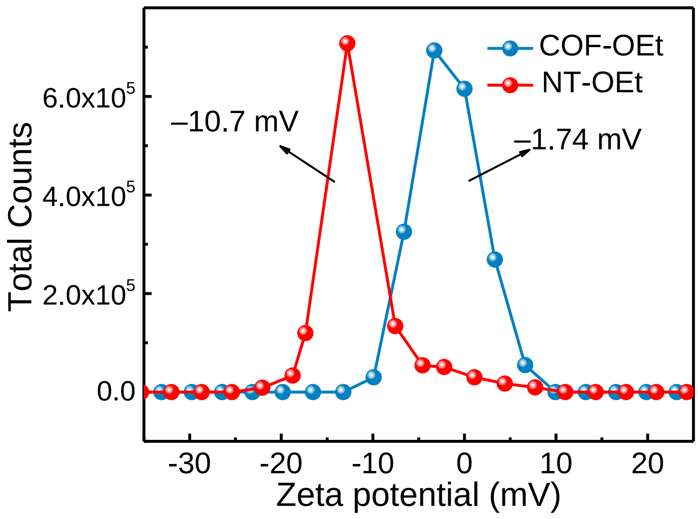

Supplementary Fig. 6 Zeta potentials of COF-OEt and NT-OEt.

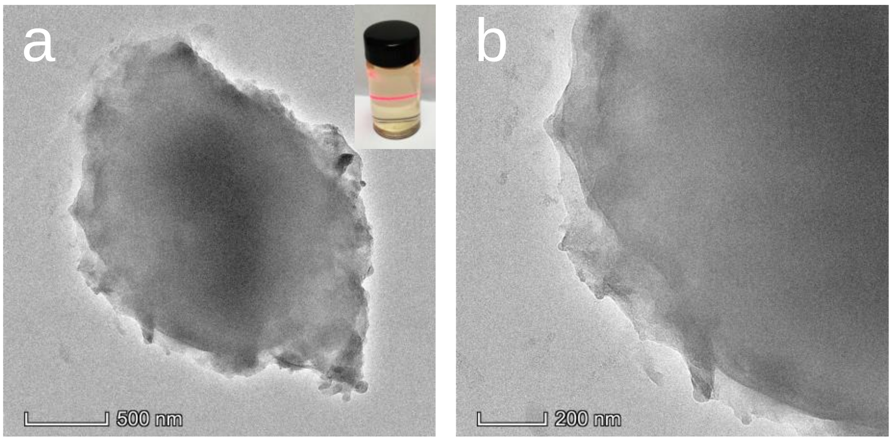

Supplementary Fig. 7 TEM images of COF-OEt with different magnifications. Scale bars: (a) 500 nm (inset image is an aqueous suspension containing COF-OEt with the Tyndall effect), (b) 200 nm.

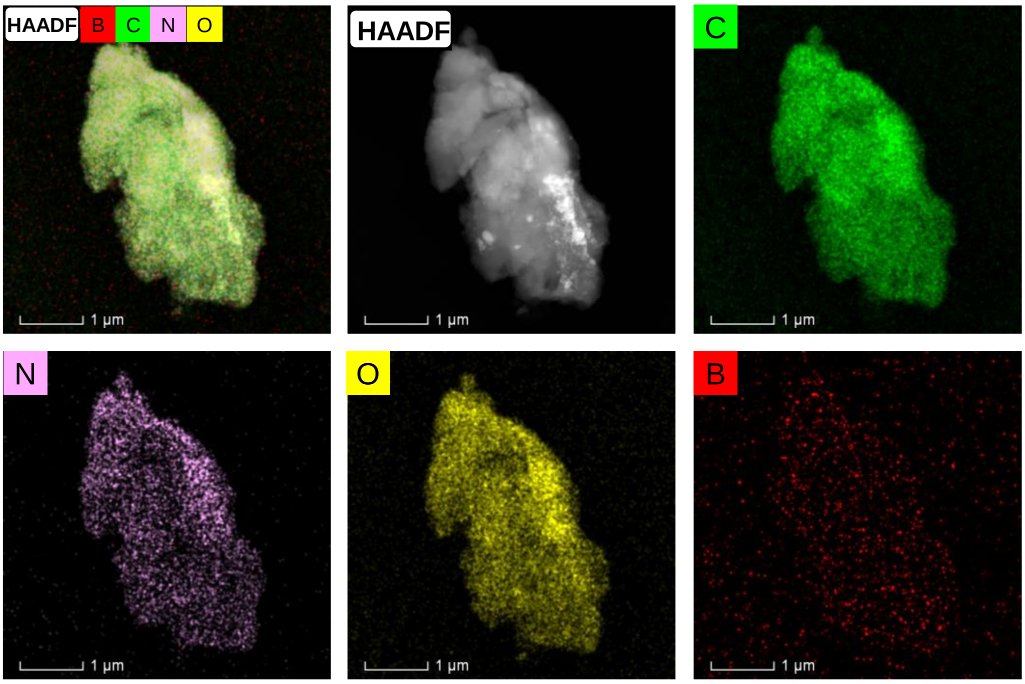

Supplementary Fig. 8 HR-TEM of COF-OEt. Scale bar: 1 µm.

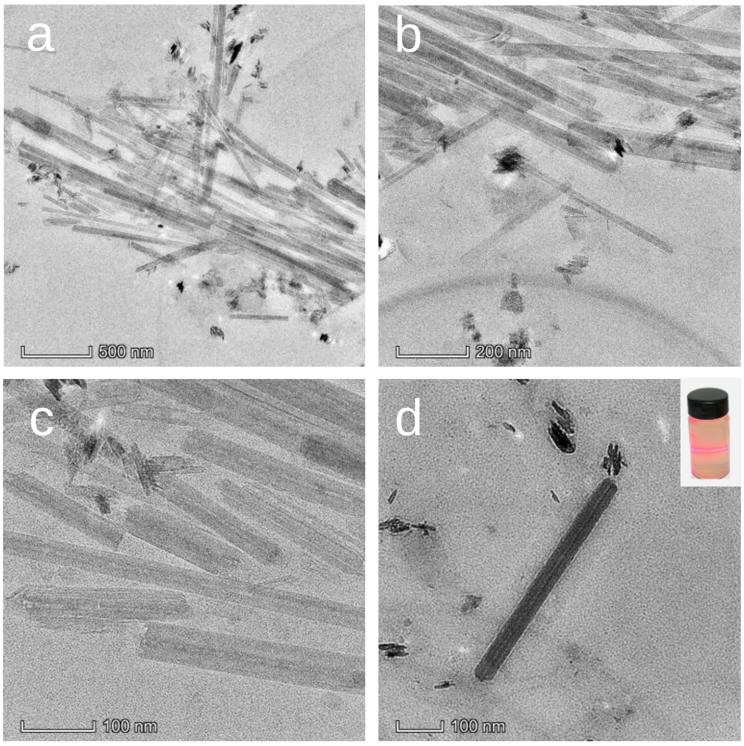

Supplementary Fig. 9 TEM images of NT-OEt. Scale bars: (a) 500 nm, (b) 200 nm, (c) 100 nm. (d) TEM image (scale bar: 100 nm) of NT-OEt at room temperature stored for 12 h (inset image is an aqueous suspension containing NT-OEt with the Tyndall effect).

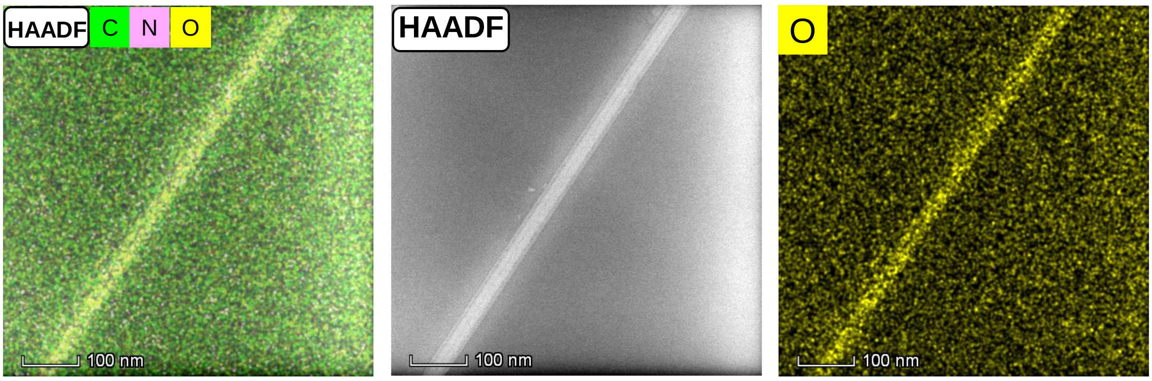

Supplementary Fig. 10 HR-TEM of a single NT-OEt. Scale bars: 100 nm.

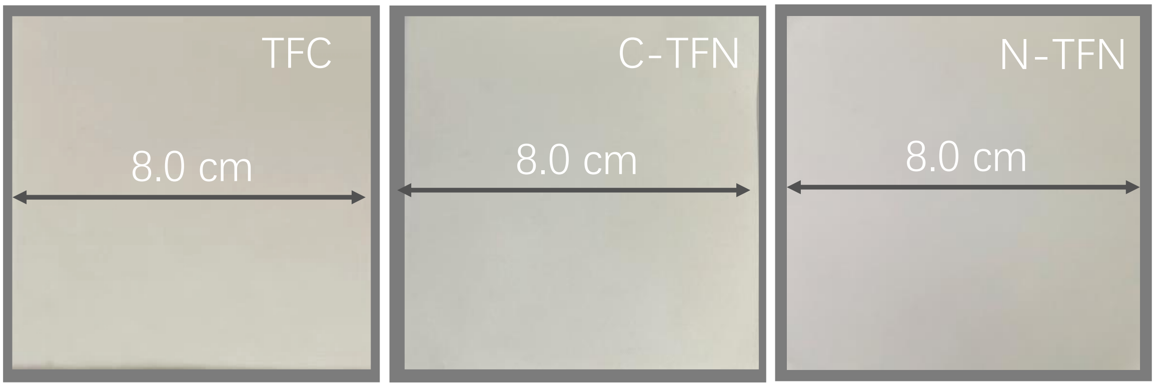

Supplementary Fig. 11 Photo images of the TFC, C-TFN and N-TFN membrane.

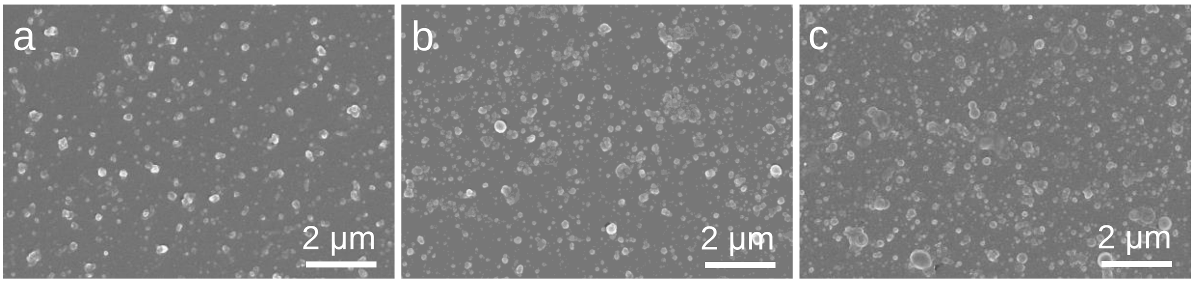

Supplementary Fig. 12 SEM images of the C-TFN membrane top surfaces, prepared with different nanoparticle loadings: (a) 1.3 μg cm^{-2}, (b) 2.6 μg cm^{-2}, (c) 3.8 μg cm^{-2}. Scale bars: 2 μm.

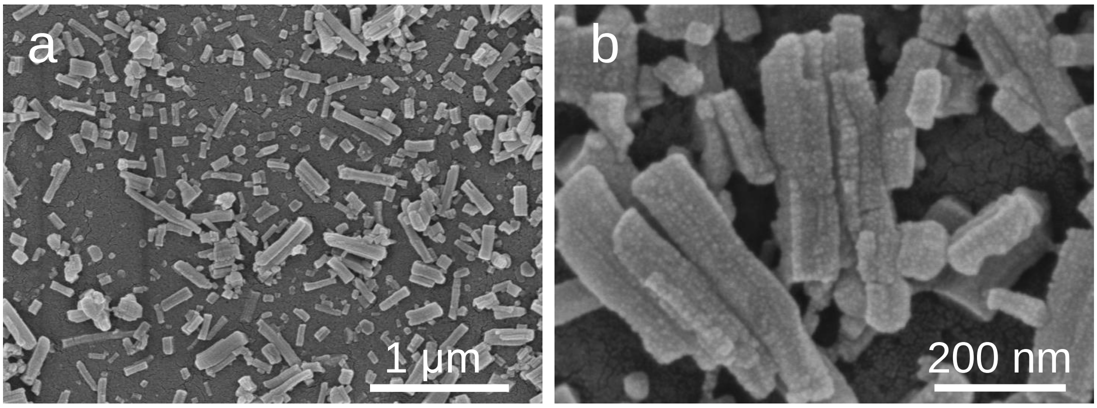

Supplementary Fig. 13 SEM images of the NT-OEt deposited membrane, prepared by loading 2.6 µg cm^{-2} of NT-OEt onto the PSf support. Scale bars: (a) 1µm, (b) 200 nm.

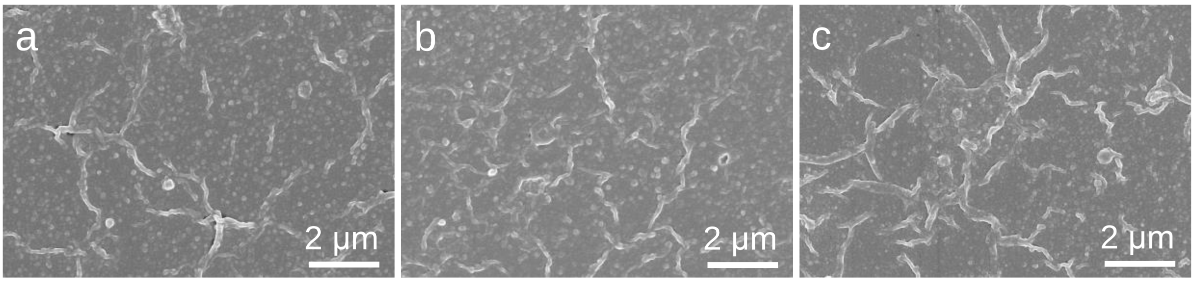

Supplementary Fig. 14 SEM images of the N-TFN membrane top surfaces, prepared with different nanoparticle loadings: (a) 0.75 μg cm^{-2}, (b) 1.3 μg cm^{-2}, (c) 3.8 μg cm^{-2}. Scale bars: 2 μm.

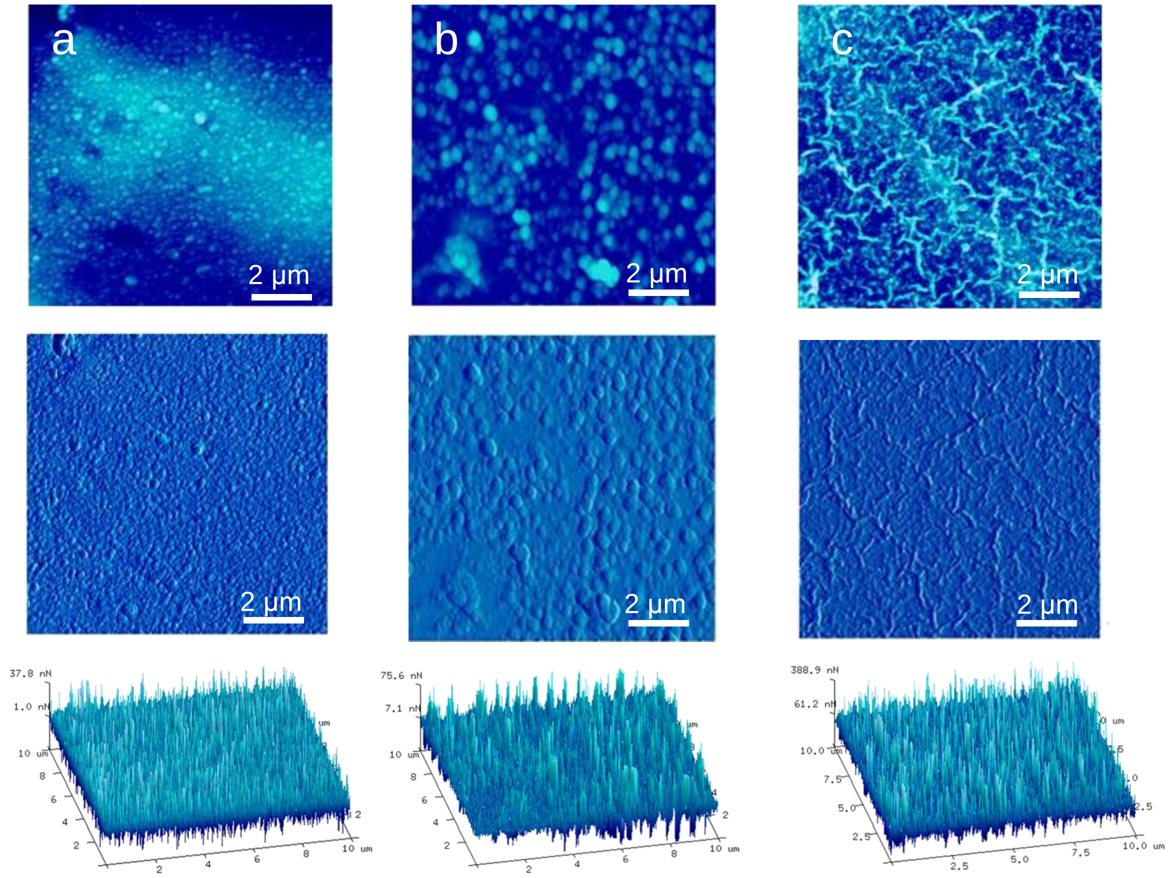

Supplementary Fig. 15 AFM images of (a) TFC, (b) C-TFN, and (c) N-TFN membranes.

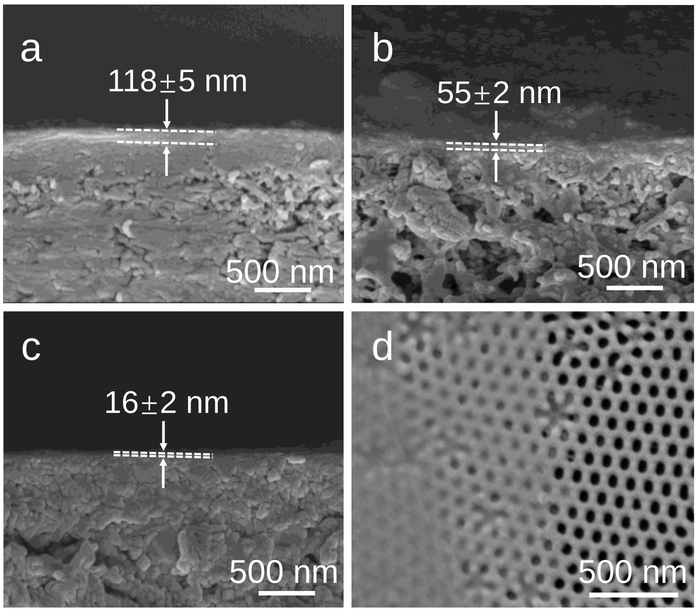

Supplementary Fig. 16 Cross-sectional SEM images of (a) TFC, (b) C-TFN, and (c) N-TFN membranes. (d) A free-standing N-TFN membrane on an anodic aluminum oxide support. Scale bars: 500 nm.

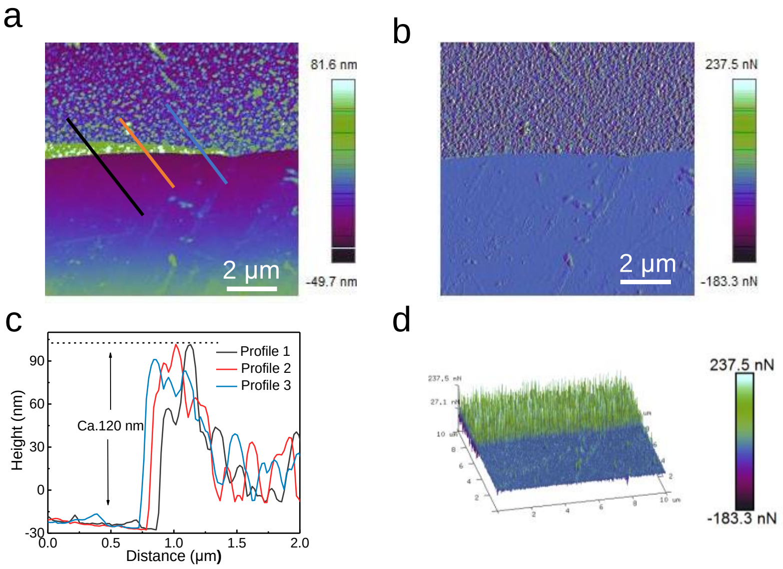

Supplementary Fig. 17 AFM images of freestanding TFC membrane: (a) height image, (b) phase image, (c) corresponding height profile, (d) 3D image.

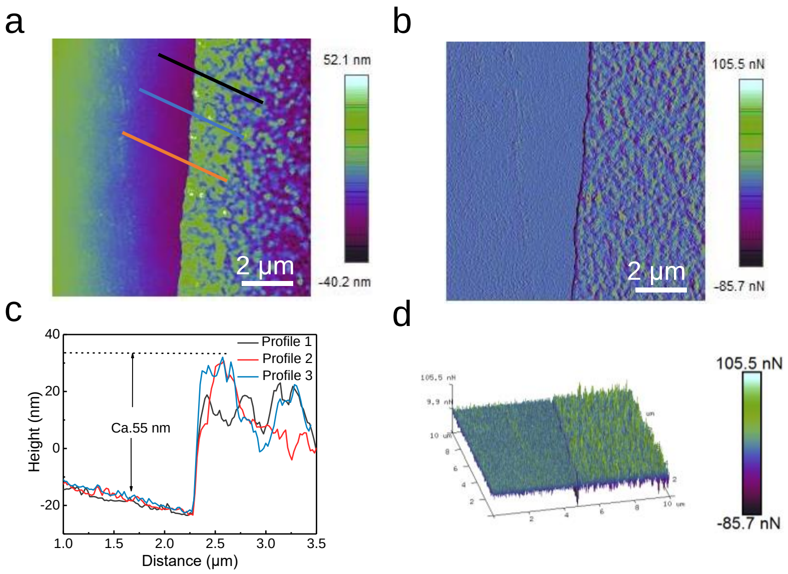

Supplementary Fig. 18 AFM images of freestanding C-TFN membrane: (a) height image, (b) phase image, (c) corresponding height profile, (d) 3D image.

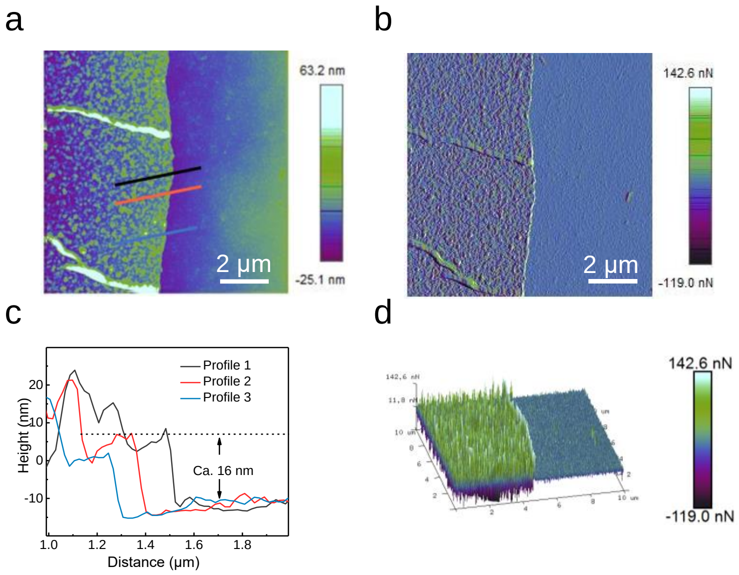

Supplementary Fig. 19 AFM images of freestanding N-TFN membrane: (a) height image, (b) phase image, (c) corresponding height profile, (d) 3D image.

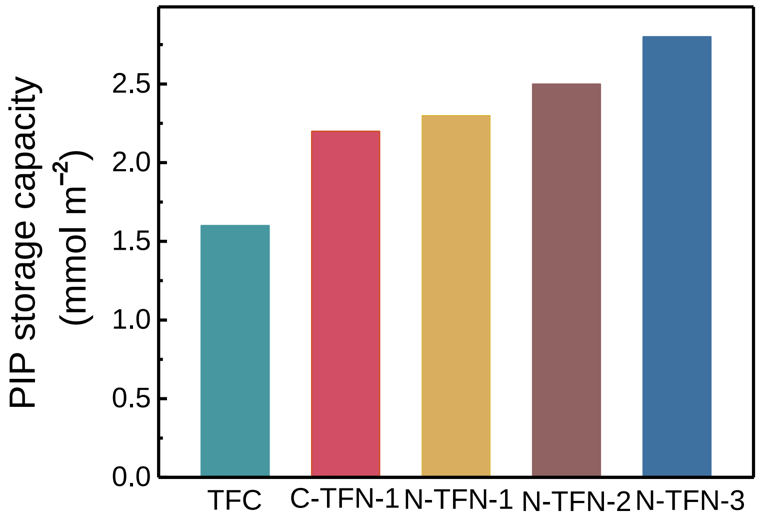

Supplementary Fig. 20 PIP storage capacity of the fabricated membranes.

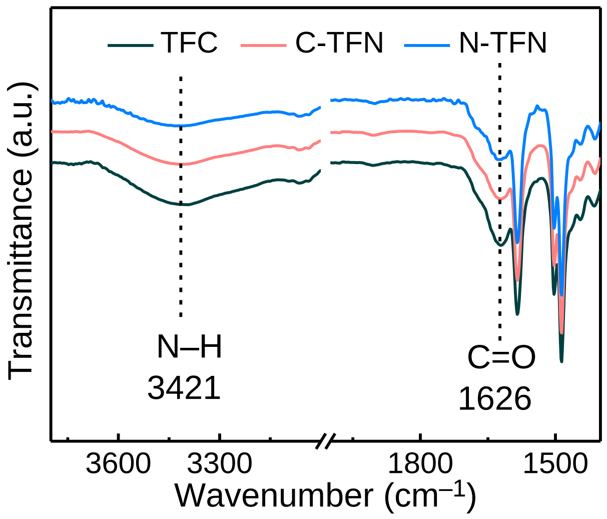

Supplementary Fig. 21 FTIR spectra of the fabricated membranes.

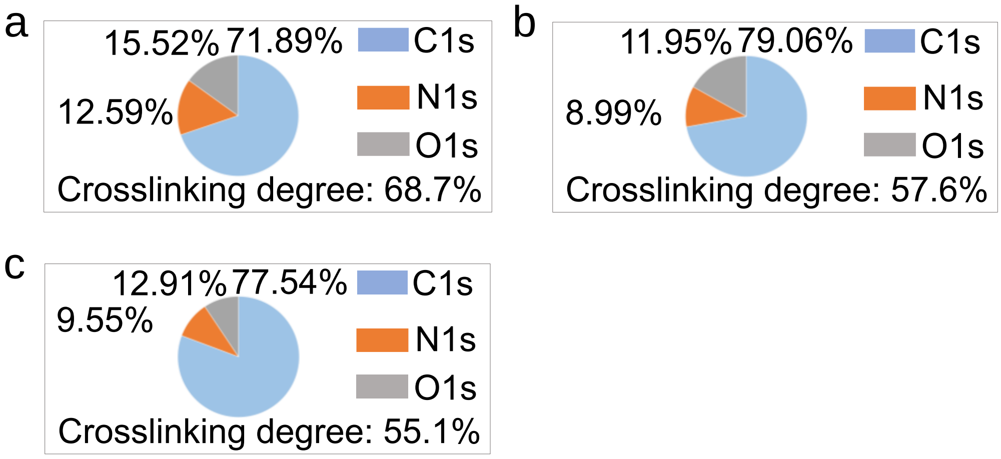

Supplementary Fig. 22 The content of C1s, N1s, O1s and degree of crosslinking of (a) TFC membrane, (b) C-TFN membrane and (c) N-TFN membrane.

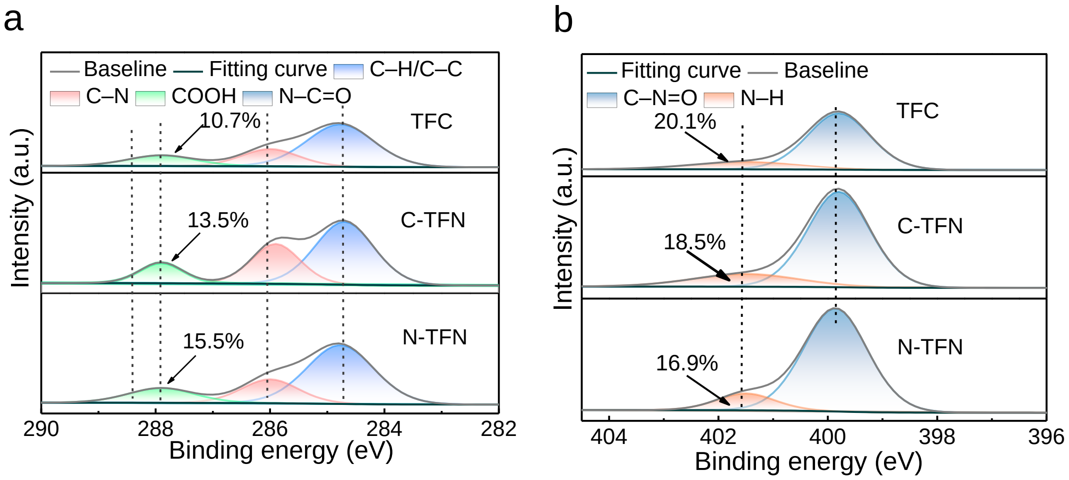

Supplementary Fig. 23 (a) C1s and (b) N1s XPS spectra of the fabricated membranes.

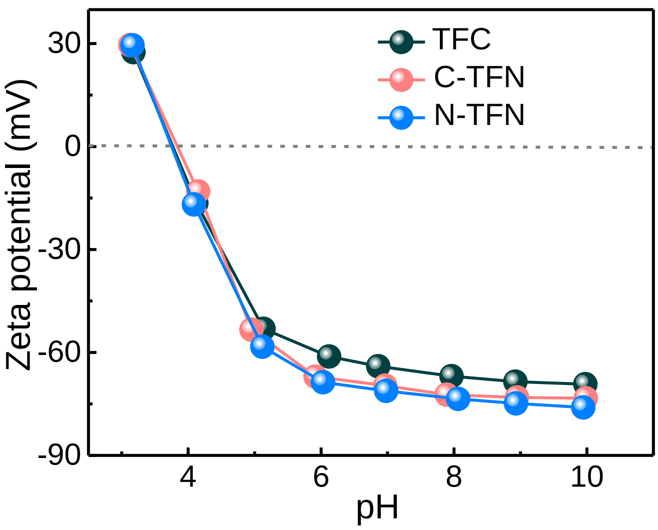

Supplementary Fig. 24 Zeta potentials as a function of pH of the fabricated membranes.

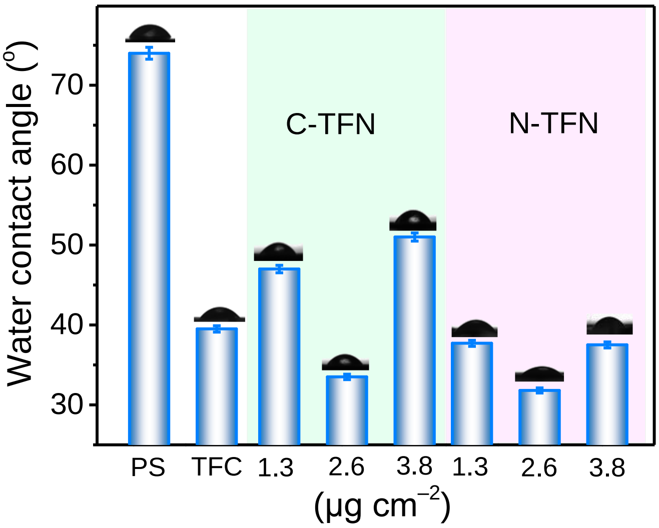

Supplementary Fig. 25 Water contact angles of as-prepared membranes.

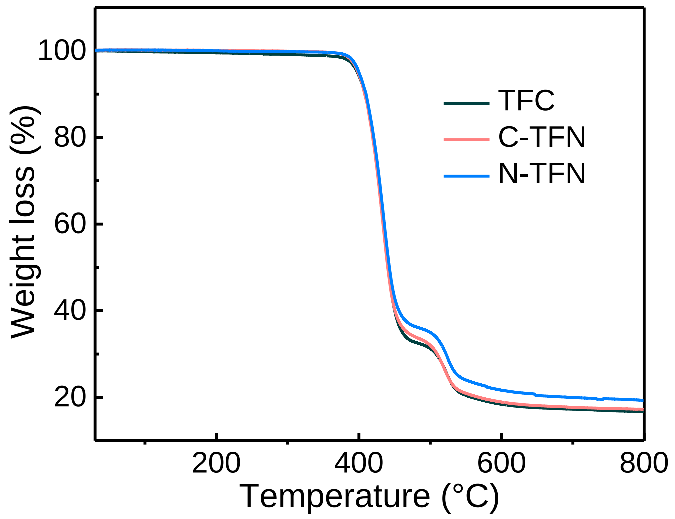

Supplementary Fig. 26 TGA decomposition curves of the fabricated membranes.

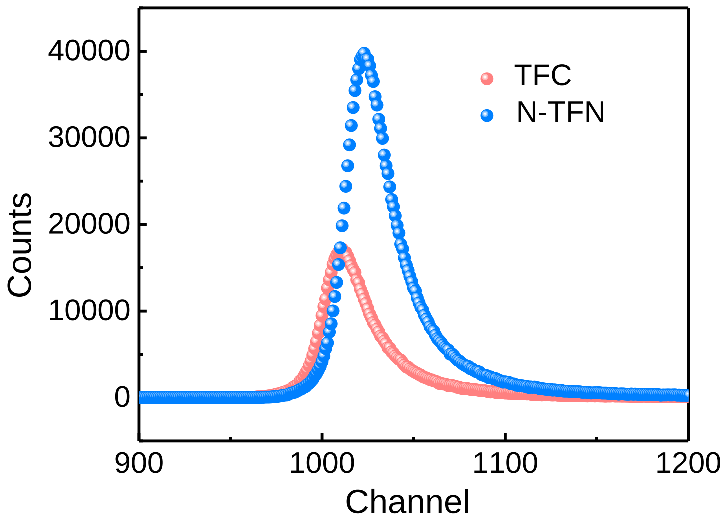

Supplementary Fig. 27 Positron annihilation patterns for the TFC and N-TFN membranes.

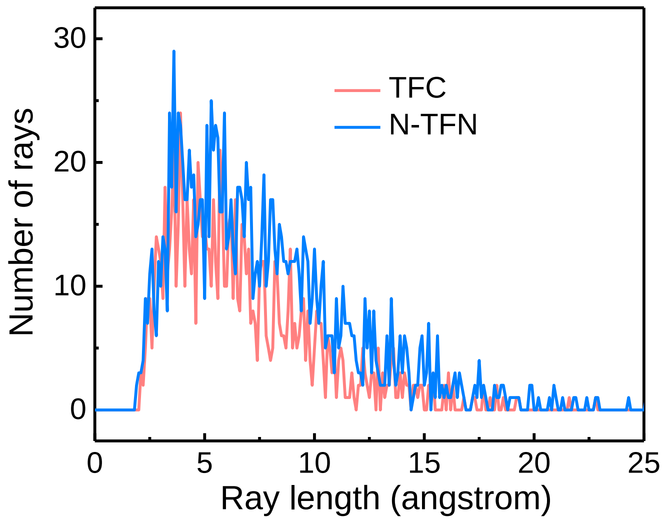

Supplementary Fig. 28 The number of rays calculated using the Zeo++ software, for the TFC and N-TFN membranes after interfacial polymerization was performed (see Section 1.6.3 of the Supporting Information for details).

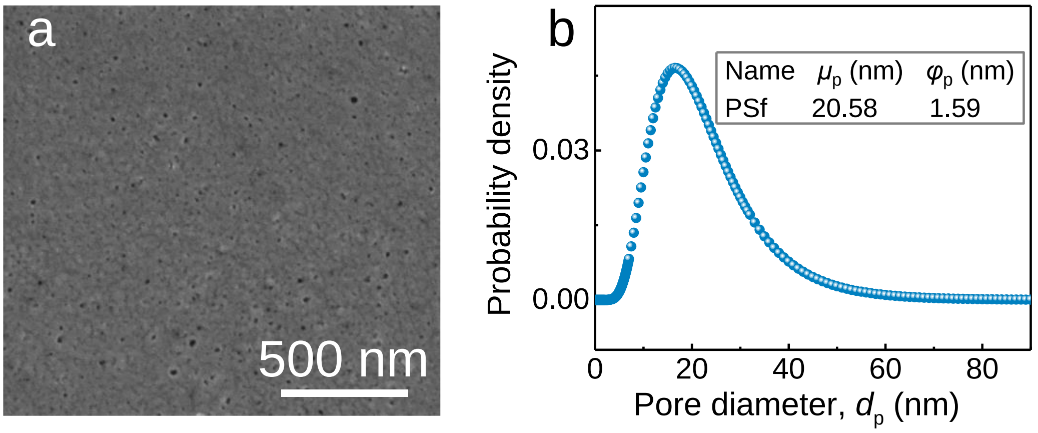

Supplementary Fig. 29 (a) SEM image and (b) pore size distribution of PSf support.

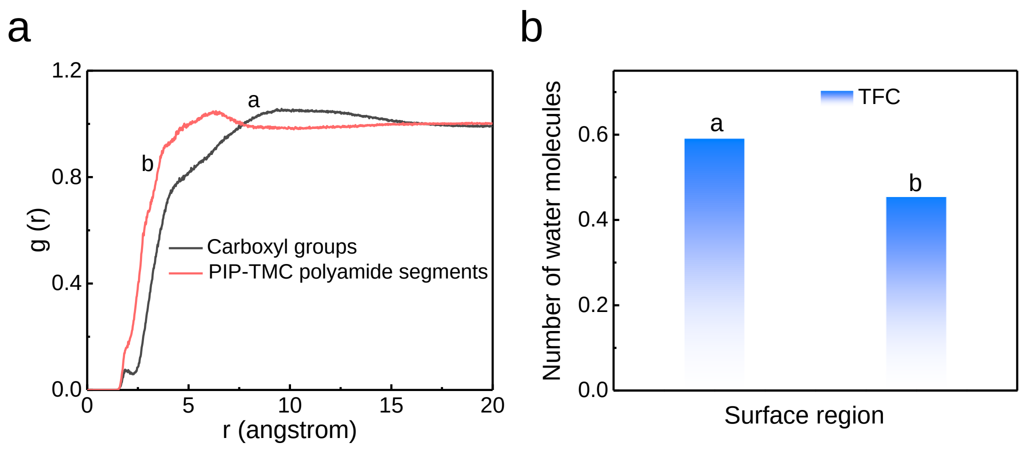

Supplementary Fig. 30 (a) Radial distribution functions between water and different segments in the TFC membrane. (b) Numbers of water molecules around the carboxyl groups (segment a) and PIP-TMC polyamide segments (segment b) in the TFC membranes, calculated from MD simulations.

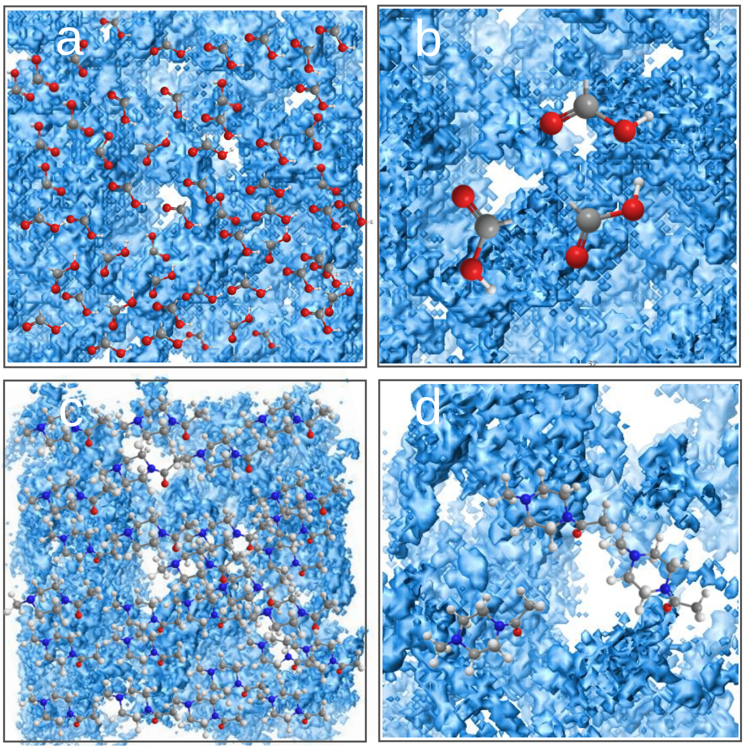

Supplementary Fig. 31 Different segments in the TFC membranes revealed by MD simulations in 100 frames. Carboxyl groups: (a) a larger version and (b) a zoomed–in version. PIP-TMC polyamide segments: (c) a larger version and (d) a zoomed–in version. The blue background refers to the distribution of water molecules in different regions of a polyamide film.

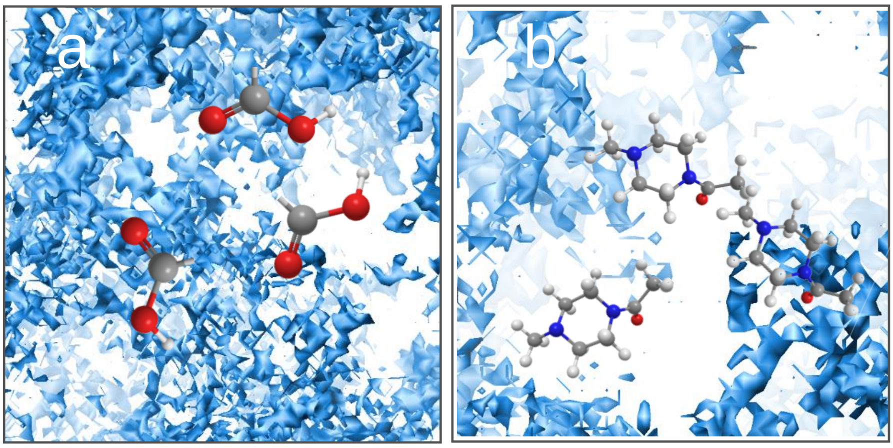

Supplementary Fig. 32 A larger version of different segments in the N-TFC membranes in 100 frames: (a) carboxyl groups, (b) PIP-TMC polyamide segments.

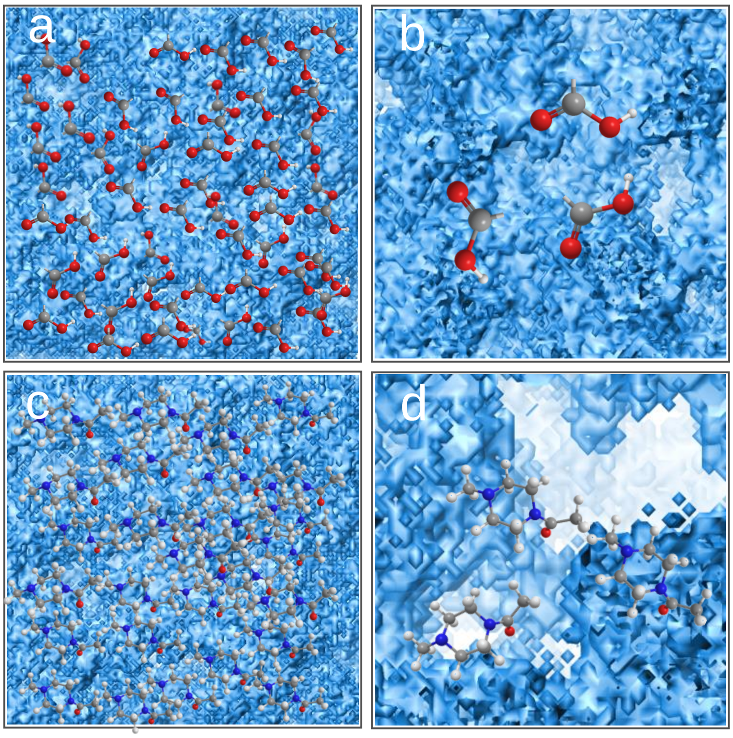

Supplementary Fig. 33 Different segments in the N-TFN membranes in 500 frames. Carboxyl groups: (a) a larger version and (b) a zoomed-in version. PIP-TMC polyamide segments: (c) a larger version and (d) a zoomed–in version.

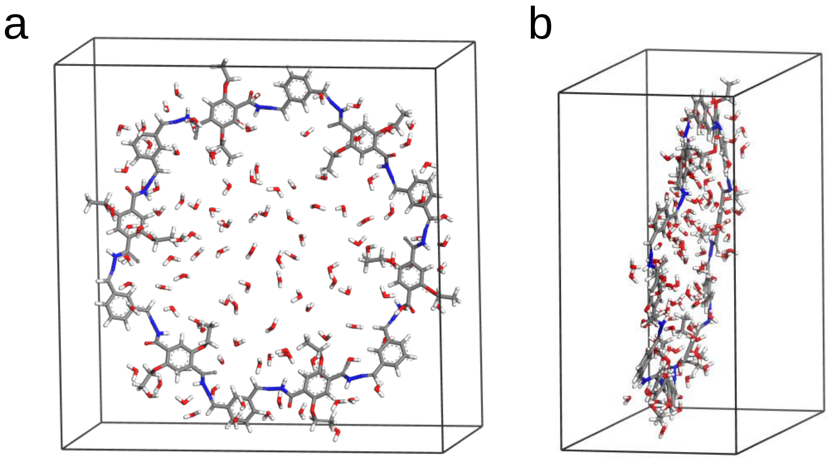

Supplementary Fig. 34 Process of water molecules passing through a nanotube: (a) front and (b) side.

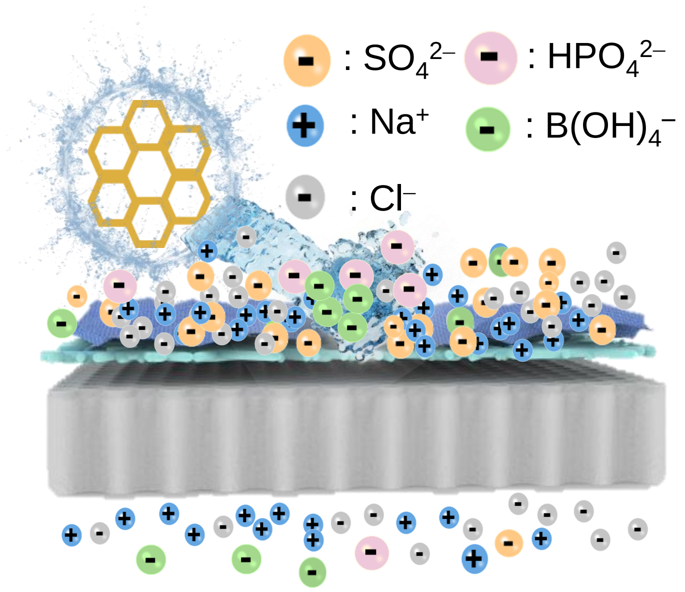

Supplementary Fig. 35 Schematic depiction of the N-TFN separation process.

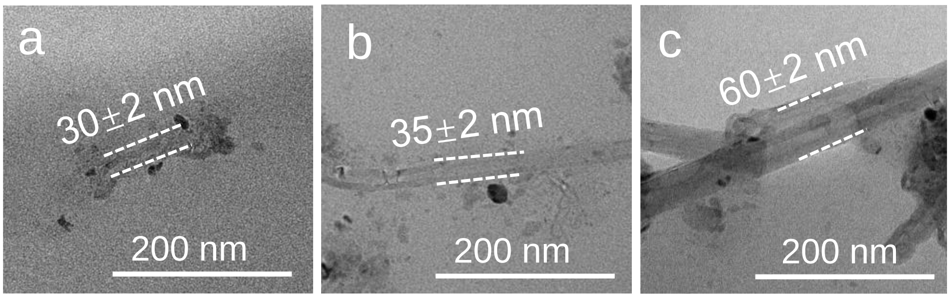

Supplementary Fig. 36 TEM images of the MONs mesh deposited in the PSf support for the different loadings (a) 1.3μg/cm^{-2}, (b) 2.6μg/cm^{-2}, (a) 3.8μg/cm^{-2}.

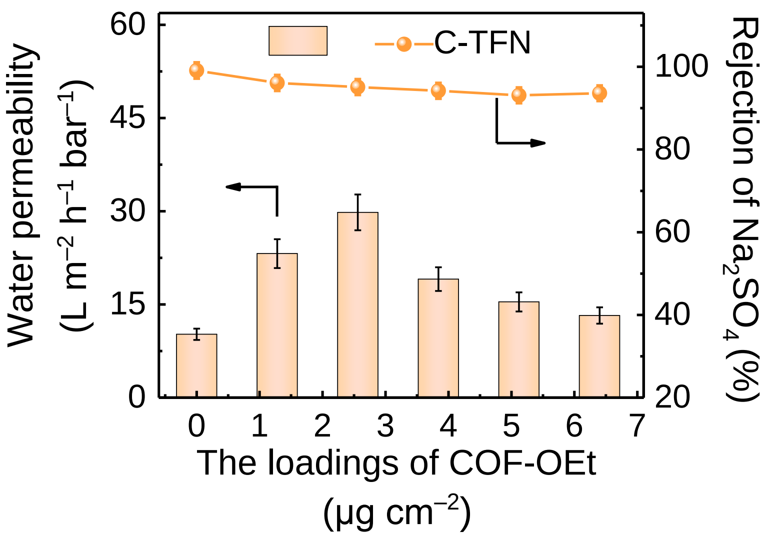

Supplementary Fig. 37 Water permeability and Na_2SO_4 rejection of the polyamide membranes fabricated with different COF-OEt contents (feed: 1000 ppm Na_2SO_4, 4 bar, pH = 7). All the error bars in this figure represent the standard deviation of the experiments.

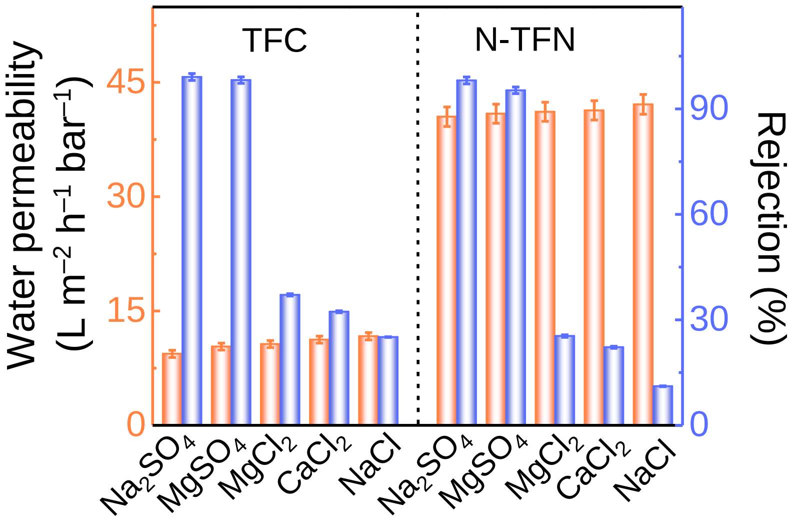

Supplementary Fig. 38 Water permeability and salt rejection of the pristine TFC membrane and N-TFN membrane for various aqueous solutions containing 1000 ppm of one inorganic salt type. All the error bars in this figure represent the standard deviation of the experiments.

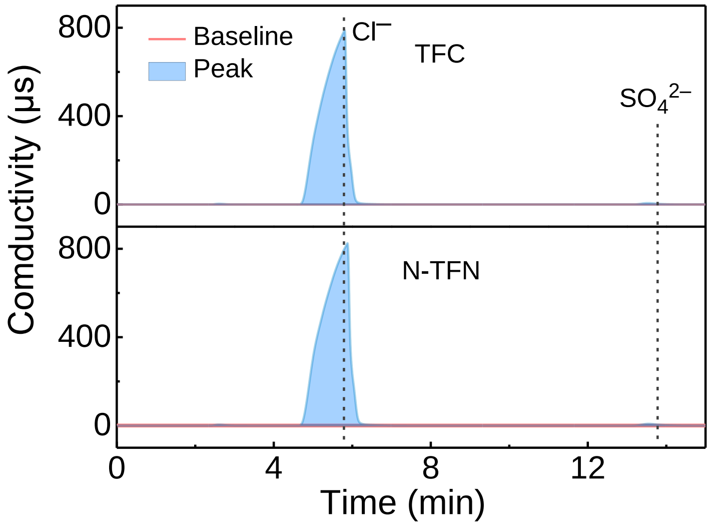

Supplementary Fig. 39 Ion chromatograph distribution curves of mixed salts.

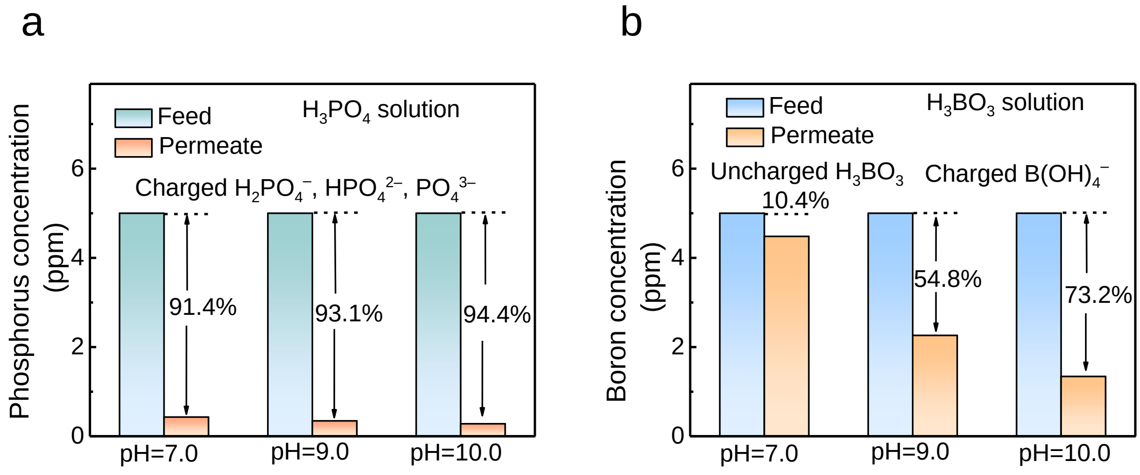

Supplementary Fig. 40 (a) Rejection of phosphorus by the TFC membranes (feed solution: 5 ppm phosphorus). (b) Rejection of boron by the TFC membranes (feed solution: 5 ppm boron). The pH was altered using a 0.1 M NaOH solution.

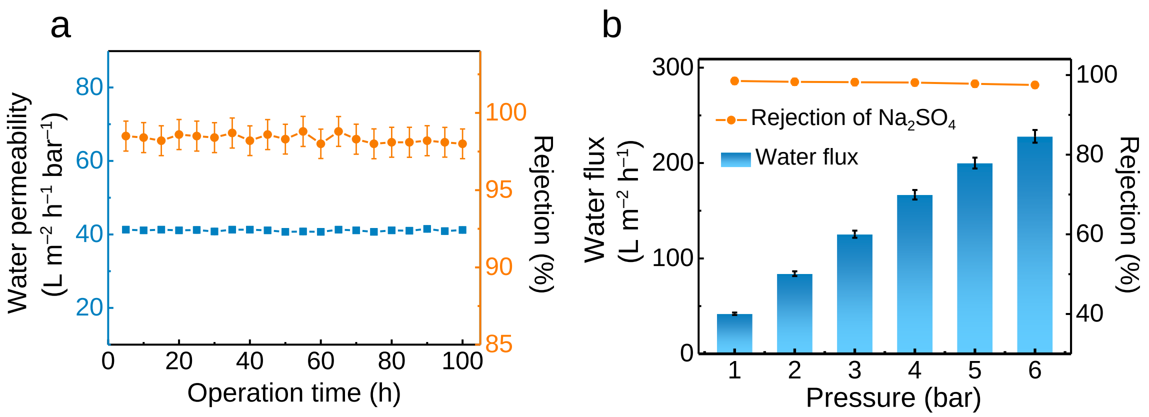

Supplementary Fig. 41 (a) Long-term stability performance of N-TFN membranes (feed: 1000 ppm Na_2SO_4). (b) Water flux and Na_2SO_4 rejection performance by N-TFN at varied pressures. All the error bars in these figures represent the standard deviation of the experiments.

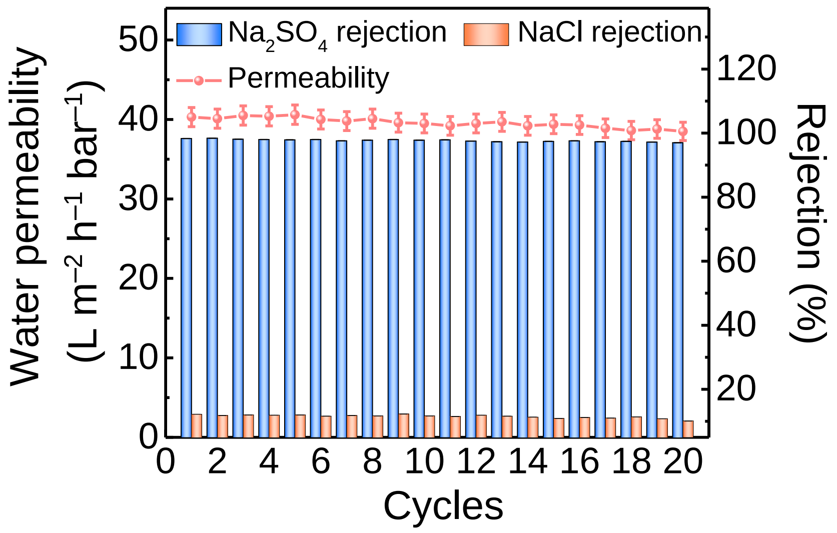

Supplementary Fig. 42 Recovery ability of the membrane of N-TFN membranes (feed: 1000 ppm Na_2SO_4 or NaCl solution, 4 bar). All the error bars in this figure represent the standard deviation of the experiments.

3. Supplementary Tables

Supplementary Table 1 Chemical species compositions of the membrane top surfaces, obtained from C1s XPS spectra.

Sample	Surface chemical species from C1s		
	B.E. (eV)	Species	Content (%)
TFC	284.9	C–H/C–C	49.8
	285.5	C–N	36.8
	287.4	COOH	10.7
	288.0	O–C=N	2.7
C-TFN	284.7	C–H/C–C	56.5
	285.9	C–N	29.6
	287.9	COOH	13.5
	288.3	O–C=N	0.4
N-TFN	284.8	C–H/C–C	62.8
	286.0	C–N	21.6
	287.9	COOH	15. 5
	288.3	O–C=N	0.1

Supplementary Table 2 Chemical species compositions of the membrane top surfaces, obtained from N1s XPS spectra.

Samples	Surface chemical species from N1s		
	B.E. (eV)	Species	Content (%)
TFC	399.6	N–C=O	79.9
	401.3	N–H	20.1
C-TFN	399.8	N–C=O	81.5
	401.5	N-H	18.5
N-TFN	399.9	N–C=O	83.1
	401.5	N–H	16.9

Supplementary Table 3 Properties of the PSf Membrane Support

Membrane ID	μ_p (nm)	σ_p (nm)	Permeance ($L\ m^{-2}\ h^{-1}\ bar^{-1}$)	MWCO (kDa)
PSf	20.58	1.59	380±30	327

Supplementary Table 4 Comparison of nanofiltration performances between the N-TFN membranes and state-of-the-art polyamide membranes.

Membranes technology	Membranes	Permeance ($L\ m^{-2}\ h^{-1}\ bar^{-1}$)	$R_{Na_2SO_4}$ (%)	R_{NaCl} (%)	$S(Cl^-/SO_4^{2-})$	Ref.
Commericial TFC	PIP@PEI-TMC	11.6	94.0	51	8.2	[12]
	Tannic Acid/Fe^{3+} TFC	13.0	97.5	65	14.0	[13]
	PAH-SP/TMC	11.3	98.2	59.2	22.7	[14]
	MPD-TFC	15.2	96.1	25.9	19	[15]
Freestanding	PEI@TMC	24.0	85.0	25.0	5.0	[16]
	COF nanosheet	6.0	98.5	35.0	43.3	[17]
	PDA@PIP-TMC	25.1	99.1	28.0	80	[18]
	LTIP@PIP-TMC	23.1	82.5	47.5	3.0	[19]
Interlayer	Macromolecular Additive/PIP-TMC	17.5	98.9	64.7	32.1	[20]
	Polydopamine interlayer TFN	19.3	90.3	20.5	8.2	[21]
	Carbon quantum dots interlayer	9.7	55.0	35.0	1.44	[22]
	Tannic	33.0	95.0	14.0	17.2	[23]

	acid/diethylenetriamine					
TFN	NaClO@PIP-BHTTM	17.5	94.0	12.0	14.7	[24]
	Hollow silica nanoparticles	15.0	92.0	11.0	11.13	[25]
	Ag NC@PIP-TMC/PEI	10.7	94.6	52.9	8.7	[26]
	Hollow Zwitterionic Nanocapsules	18.7	95.6	58.2	9.5	[27]
Lab made TFC	DAPP-TMC	6.6	95.0	40.0	12.0	[28]
	SDS/PIP-TMC	12.6	97.1	15.1	29.3	[29]
	EIP@PIP-TMC	20.2	27.0	6.3	1.3	[30]
	DEEDA@PIP-TMC	14.6	96.6	18.5	24.0	[31]
CNT TFN	CNT-TFN	13.0	96.5	24.6	21.5	[32]
	TFNi	29.6	97.0	23.0	25.7	[33]
	MWCNTs interlayer TFN	6.2	96.6	34.0	19.4	[34]
	MWCNTs@PEI-TMC	15.3	45.2	33.8	1.21	[35]
MOF TFN	UiO-66-NH_2-TFN	18.0	98.4	30.2	43.6	[36]

	UiO-66-TFN	11.5	91.0	38.0	6.9	[37]
	PSS modified ZIF-8	12.8	99.0	15.0	85	[38]
	PDA/MOF-101@MPD-TMC	2.12	99.0	93.5	6.5	[39]
COF TFN	Polydopamine-COF interlayer	16.4	97.2	14.6	30.5	[40]
	TpPa COFs	21.0	98.4	21.5	49.0	[41]
	Amine-rich COFs	19.3	83.5	18.0	5.0	[42]
	COF nanofibers	31.1	95.0	16.0	16.8	[43]
N-TFN	CNT-TFNi	18.9	98.0	17.9	41.1	This work
	NT-OEt/PIP-TMC	41.7	98.1	11	46.8	

Supplementary Table 5 Summarized boron removal performance of state-of-the-art NF membrane desalination technology.

Membranes	Permeance (L m^{-2} h^{-1} bar^{-1})	Concentration (ppm)	pH	R_{Boron} (%)	Ref.
CK-NF	7.05	5.7	8.4	26.0	[44]
NF90	3.6	5.7	8.4	57.0	[44]
	~7	50	7	~ 40	
	~6	50	9	~60	[45]
	~6	50	11	~95	
	~10.5	4.6	6	~10	
		4.6	7	~20	
		4.6	8	~11	[46]
		4.6	9	~30	
		4.6	10	~50	
		4.6	11	~80	
	~ 5.5	3	3	~15	
		3	5	~10	
		3	7	~16	[47]
		3	9	~20	
		3	11	~90	
		3	12.5	~95	

NF270	12.7	5.7	8.4	66.7	[44]
	~13	50	7	~10	
	~12	50	9	~20	[45]
	~15	50	11	~70	
	~14.0	4.6	6	~5	
		4.6	7	~6	
		4.6	8	~10	[46]
		4.6	9	~25	
		4.6	10	~35	
		4.6	11	~55	
TFC-S	~ 2.9	3	3	~9	
		3	5	~10	
		3	7	~8	[47]
		3	9	~15	
		3	11	~75	
		3	12.5	~85	
UTC-60	~ 16.4	3	3	~ 5	
		3	5	~3	
		3	7	~4	[47]
		3	9	~2	
		3	11	~25	
		3	12.5	~50	

ESPA4	~ 3.2	3	3	~27	
		3	5	~28	
		3	7	~29	
		3	9	~30	[47]
		3	11	~80	
		3	12.5	~98	
N-L1.5	4.0	10	10	83.6	[48]
P84-HPEI-SCA4 TFN	3.91	10	4	55.4	
		10	7	67.2	
		10	8	87.5	[49]
		10	9	90.0	
		10	10	98.2	
PT-TFC	~10.5	2000	7	~58	
MSH-TFN	~9.5	2000	7	~62	[50]
MSH@UiO-66-NH2-TFN	~8.5	2000	7	71.2	
M-1.0TEA	1.44	~	8.1	40.0	[51]
NF-200	3.82	~	8.1	16.8	
TFC	9.8	5	7	10.4	
		5	9	54.8	
		5	10	73.2	This

N-TFN	41.7	5	7	11.0	work
		5	9	65.1	
		5	10	78.0	

Supplementary Table 6 Summarized phosphorus removal performance of state-of-the-art NF membrane desalination technology.

Membranes	Permeance ($L\ m^{-2}\ h^{-1}\ bar^{-1}$)	Concentration (ppm)	pH	$R_{phosphorus}$ (%)	Ref.
La/C-doped	—	20	5	~70	
		20	7	~80	
		20	9	>90	[52]
		20	11	>90	
N-TFN	41.7	5	7	93.2	
		5	9	93.4	This work
		5	10	96.8	

Supplementary References

1. Tang, C., Kwon, Y. & Leckie, J. Probing the nano– and micro-scales of reverse osmosis membranes-A comprehensive characterization of physiochemical properties of uncoated and coated membranes by XPS, TEM, ATR-FTIR, and streaming potential measurements. *J. Membr. Sci.* **287**, 146–156 (2007).
2. Akin, O. & Temelli, F. Probing the hydrophobicity of commercial reverse osmosis membranes produced by interfacial polymerization using contact angle, XPS, FTIR, FE-SEM and AFM. *Desalination* **278**, 387–396 (2011).
3. Han, G., Chung, T-S. & Toriida, M. Thin-film composite forward osmosis membranes with novel hydrophilic supports for desalination, *J. Membr. Sci.* **423**, 543–555 (2012).
4. Ma, D., Peh, S. B., Han, G. & Chen, S. B. Thin-Film Nanocomposite (TFN) Membranes Incorporated with Super–Hydrophilic Metal–Organic Framework (MOF) UiO-66: Toward Enhancement of Water Flux and Salt Rejection. *ACS Appl. Mater. Interfaces* **9**, 7523–7534 (2017).
5. Yao, L., Qin, Z., Chen, Q., Zhao, M., Zhao, H., Ahmad, W., Fan, L. & Zhao, L. Insights into the nanofiltration separation mechanism of monosaccharides by molecular dynamics simulation, *Sep. Purif. Technol.* **205**, 48–57 (2018).
6. Willems, T. F., Rycroft, C. H., Kazi, M., Meza, J. C. & Haranczyk, M. Algorithms and tools for high-throughput geometry-based analysis of crystalline porous materials, *Micropor. Mesopor. Mat.* **149**, 134–141 (2012).
7. Pinheiro, M., Martin, R. L., Rycroft, C. H., Jones, A., Iglesia, E. & Haranczyk, M.

Pore size distributions and stochastic ray tracing: Characterization and comparison of pore landscapes in crystalline porous materials, *J. Mol. Graph. Model.* **44**, 208–219 (2013).

8. Pinheiro, M., Martin, R. L., Rycroft, C. H., Jones, A., Iglesia, E. & Haranczyk, M. High-accuracy Voronoi calculations: High accuracy geometric analysis of crystalline porous materials, *Cryst. Eng. Comm.* **15**, 7531–7538 (2013).

9. Bethel, E. W. et al. High performance visualization: Enabling extreme-scale scientific insight and Life Ch. 16 (CRC Press, New York, 2012).

10. Robertson, M. J., Tirado-Rives, J. & Jorgensen, W. L. Improved peptide and protein torsional energetics with the OPLS-AA force field. *J. Chem. Theory Comput.* **11**, 3499–3509 (2015).

11. Jorgensen, W. L., Maxwell, D. S. & Tirado-Rives, J. Development and testing of the OPLS all-atom force field on conformational energetics and properties of organic liquids. *J. Am. Chem. Soc.* **118**, 11225–11236 (1996).

12. Wu, D., Yu, S., Lawless, D. & Feng, X. Thin film composite nanofiltration membranes fabricated from polymeric amine polyethylenimine imbedded with monomeric amine piperazine for enhanced salt separations. *React. Funct. Polym.* **86**, 168–183 (2015).

13. Yang, Z., Zhou, Z. W., Guo, H., Yao, Z., Ma, X. H., Song, X., Feng, S. P. & Tang, C. Y. Tannic acid/Fe^{3+} nanoscaffold for interfacial polymerization: toward enhanced nanofiltration performance. *Environ. Sci. Technol.* **52**, 9341–9349 (2018).

14. Jeon, S., Park, C. H., Park, S. H., Shin, M. G., Kim, H. J., Baek, K. Y., Chanc, P. E.

& Lee, J. H. Star polymer-assembled thin film composite membranes with high separation performance and low fouling. *J. Membr. Sci.* **555**, 369–378 (2018).

15. Li, Y., Wong, E., Mai, Z. & Van der Bruggen, B. Fabrication of composite polyamide/Kevlar aramid nanofiber nanofiltration membranes with high permselectivity in water desalination. *J. Membr. Sci.* **592**, 117396, (2019).
16. Trivedi, J. S., Bhalani, D. V., Bhadu, G. R. & Jewrajka, S. K. Multifunctional amines enable the formation of polyamide nanofilm composite ultrafiltration and nanofiltration membranes with modulated charge and performance. *J. Mater. Chem. A* **6**, 20242–20253 (2018).
17. Zhang, Z., Yin, C., Yang, G., Xiao, A., Shi, X., Xing, W. & Wang, Y. Stitching nanosheets of covalent organic frameworks to build aligned nanopores in nanofiltration membranes for precise ion separations. *J. Membr. Sci.* **618**, 118754 (2021).
18. Zhu, J., Hou, J., Zhang, R., Yuan, S., Li, J., Tian, M., Wang, P., Zhang, Y., Volodin, A. & Van der Bruggen, B. Rapid water transport through controllable, ultrathin polyamide nanofilms for high-performance nanofiltration. *J. Mater. Chem. A* **6**, 15701–15709 (2018).
19. Liu, S., Wu, C., Hung, W-S., Lu, X. & Lee, K-R. One-step constructed ultrathin Janus polyamide nanofilms with opposite charges for highly efficient nanofiltration. *J. Mater. Chem. A* **5**, 22988–22996 (2017).
20. Yang, X. Monitoring the interfacial polymerization of piperazine and trimesoyl chloride with hydrophilic interlayer or macromolecular additive by in situ FT-IR

spectroscopy. *Membr.* **10**, 12 (2020).

21. Yang, Z., Wang, F., Guo, H., Peng, L. E., Ma, X. H., Song, X. X., Wang, Z. W. & Tang, C. Y. Mechanistic insights into the role of polydopamine interlayer toward improved separation performance of polyamide nanofiltration membranes. *Environ. Sci. Technol.* **54**, 11611–11621 (2020).
22. Yang, W-J., Shao, D-D., Zhou, Z., Xia, Q. C., Chen, J., Cao, X. L., Zheng, T. & Sun, S. P. Carbon quantum dots (CQDs) nanofiltration membranes towards efficient biogas slurry valorization. *Chem. Eng. J.* **385**, 123993 (2020).
23. Zhang, X., Liu, C., Yang, J., Zhu, C-Y., Zhang, L. & Xu, Z-K. Nanofiltration membranes with hydrophobic microfiltration substrates for robust structure stability and high water permeation flux. *J. Membr. Sci.* **593**, 117444 (2020).
24. Tang, Y-J., Wang, L-J., Xu, Z-L., Wei, Y-M. & Yang, H. Novel high-flux thin film composite nanofiltration membranes fabricated by the NaClO pre-oxidation of the mixed diamine monomers of PIP and BHTTM in the aqueous phase solution. *J. Membr. Sci.* **502**, 106–115 (2016).
25. Yap Ang, MBM., Huang, S-H., Tsai, S-J., De Guzman, MR., Lee, K-R. & Lai, J-Y. Embedding hollow silica nanoparticles of varying shapes and dimensions in nanofiltration membranes for optimal performance. *J. Membr. Sci.* **611**, 118333, (2020).
26. Bera, A., Trivedi, JS., Kumar, SB., Chandel, AKS., Haldar, S. & Jewrajka, SK. Anti-organic fouling and anti-biofouling poly(piperazineamide) thin film nanocomposite membranes for low pressure removal of heavy metal ions. *J. Hazard.*

Mater. **343**, 86–97 (2018).

27. Sun, Z., Wu, Q., Ye, C., Wang, W., Zheng, L., Dong, F., Yi, Z., Xue, L. & Gao, C. Nanovoid Membranes Embedded with Hollow Zwitterionic Nanocapsules for a Superior Desalination Performance. *Nano Lett.* **19**, 2953–2959 (2019).
28. Veríssimo, S., Peinemann, K. V. & Bordado, J. Influence of the diamine structure on the nanofiltration performance, surface morphology and surface charge of the composite polyamide membranes. *J. Membr. Sci.* **279**, 266–275 (2006).
29. Liang, Y., Zhu, Y., Liu, C., Lee, K. R., Hung, W. S., Wang, Z., Li, Y., Elimelech, M. & Jin, J., Lin, S. Polyamide nanofiltration membrane with highly uniform sub-nanometre pores for sub–1 Å precision separation. *Nat. Commun.* **11**, 1–9 (2020).
30. Kang, Y., Jang, J., Kim, S., Lim, J., Lee, Y. & Kim, IS. PIP/TMC Interfacial Polymerization with Electrospray: Novel Loose Nanofiltration Membrane for Dye Wastewater Treatment. *ACS Appl. Mater. Interfaces* **12**, 36148–36158 (2020).
31. Guo, Y-S., Ji, Y-L., Wu, B., Wang, N-X., Yin, M-J., An, Q-F. & Gao, C-J., High-flux zwitterionic nanofiltration membrane constructed by in-situ introduction method for monovalent salt/antibiotics separation. *J. Membr. Sci.* **593**, 117441, (2020).
32. Long, L., Wu, C., Yang, Z. & Tang, C. Y. Carbon nanotube interlayer enhances water permeance and antifouling performance of nanofiltration membranes: mechanisms and experimental evidence. *Environ. Sci. Technol.* **56**, 2656–2664 (2022).
33. Liu, C., Wang, W., Zhu, L., Cui, F., Xie, C., Chen, X. & Li, N. High–performance

nanofiltration membrane with structurally controlled PES substrate containing electrically aligned CNTs. *J. Membr. Sci.* **605**, 118104 (2020).

34. Xue, S.-M., Xu, Z.-L., Tang, Y.-J. & Ji, C.-H. Polypiperazine-amide nanofiltration membrane modified by different functionalized multiwalled carbon nanotubes (MWCNTs). *ACS Appl. Mater. Interfaces* **8**, 19135–19144 (2016).
35. Zhao, F. Y., Ji, Y. L., Weng, X. D., Mi, Y. F., Ye, C. C., An, Q. F. & Gao, C. J. High-Flux Positively Charged Nanocomposite Nanofiltration Membranes Filled with Poly(dopamine) Modified Multiwall Carbon Nanotubes. *ACS Appl. Mater. Interfaces* **8**, 6693–6700 (2016).
36. Zhu, J., Hou, J., Yuan, S., Zhao, Y., Li, Y., Zhang, R., Tian, M., Li, J., Wang, J. & Van der Bruggen, B. MOF-positioned polyamide membranes with a fishnet-like structure for elevated nanofiltration performance. *J. Mater. Chem. A* **7**, 16313–16322 (2019).
37. He, Y., Tang, YP., Ma, D. & Chung, T-S. UiO-66 incorporated thin-film nanocomposite membranes for efficient selenium and arsenic removal. *J. Membr. Sci.* **541**, 262–270 (2017).
38. Zhu, J., Qin, L., Uliana, A., Hou, J., Wang, J., Zhang, Y., Lin, X., Yuan, S., Li, J., Tian, M., Lin, J. & Van der Bruggen, B. Elevated Performance of Thin Film Nanocomposite Membranes Enabled by Modified Hydrophilic MOFs for Nanofiltration. *ACS Appl. Mater. Interfaces* **9**, 1975–1986 (2017).
39. He, M., Wang, L., Lv, Y., Wang, X., Zhu, J., Zhang, Y. & Liu, T. Novel polydopamine/metal organic framework thin film nanocomposite forward osmosis

membrane for salt rejection and heavy metal removal. *Chem. Eng. J.* **389**, 124452, (2020).

40. Wu, M., Yuan, J., Wu, H., Su, Y., Yang, H., You, X., Zhang, R., He, X., Khan, N., Kasher, R. & Jiang, Z. Ultrathin nanofiltration membrane with polydopamine-covalent organic framework interlayer for enhanced permeability and structural stability. *J. Membr. Sci.* **576**, 131–141 (2019).
41. Zhang, T., Li, P., Ding, S. & Wang, X. High permeability composite nanofiltration membrane assisted by introducing TpPa covalent organic frameworks interlayer with nanorods for desalination and NaCl/dye separation. *Sep. Purif. Technol.* **270**, 118802 (2021).
42. Wang, C., Li, Z., Chen, J., Li, Z., Yin, Y., Cao, L., Zhong, Y. & Wu, H. Covalent organic framework modified polyamide nanofiltration membrane with enhanced performance for desalination. *J. Membr. Sci.* **523**, 273–281 (2017).
43. Zhang, Z., Shi, X., Wang, R., Xiao, A. & Wang, Y. Ultra-permeable polyamide membranes harvested by covalent organic framework nanofiber scaffolds: a two-in-one strategy. *Chem. Sci.* **10**, 9077–9083 (2019).
44. Jarma, Y. A., Karaoğlu, A., Tekin, Ö., Baba, A., Ökten, H. E., Tomaszewska, B., Kamil, B., Müserref, A. & Kabay, N. Assessment of different nanofiltration and reverse osmosis membranes for simultaneous removal of arsenic and boron from spent geothermal water. *J. Hazard. Mater.* **405**, 124129 (2021).
45. Tagliabue, M., Bagatin, R., Capannelli, G., Cattaneo, C., Bottino, A., Firpo, R., Jezowska, A. & Voena, A. Multivariate comparison of reverse osmosis and

nanofiltration membranes through tree cluster analysis. *Desalin. Water Treat.* **57**, 23273–23279 (2015).

46. Tu, K. L., Nghiem, L. D. & Chivas, A. R. Coupling effects of feed solution pH and ionic strength on the rejection of boron by NF/RO membranes. *Chem. Eng. J.* **168**, 700–706 (2011).
47. Richards, L. A., Vuachère, M. & Schäfer, A. I. Impact of pH on the removal of fluoride, nitrate and boron by nanofiltration/reverse osmosis. *Desalination* **261**, 331–337 (2010).
48. Ghiasi, S., Mohammadi, T. & Tofighy, M. A. Hybrid nanofiltration thin film hollow fiber membranes with adsorptive supports containing bentonite and LDH nanoclays for boron removal. *J. Membr. Sci.* **655**, 120576 (2022).
49. Abdi, Z.G., Chen, J-C. & Chung, T-S. Infiltration of 3D-macrocycles to integrally skinned asymmetric P84 co-polyimide membranes for boron removal. *Desalination* **540**, 115988 (2022).
50. Gohain, M.B., Pawar, R.R., Karki, S., Hazarika, A., Hazarika, S. & Ingole, P.G. Development of thin film nanocomposite membrane incorporated with mesoporous synthetic hectorite and MSH@UiO-66-NH_2 nanoparticles for efficient targeted feeds separation, and antibacterial performance. *J. Membr. Sci.* **609**, 118212 (2020).
51. Kumar, R., Ahmed, M., Ok, S., Garudachari, B. & Thomas, J. P. Boron selective thin film composite nanofiltration membrane fabricated via a self-assembled trimesic acid layer at a liquid–liquid interface on an ultrafiltration support. *New J. Chem.* **43**, 3874–3883 (2019).

52. Xia, W.-J., Guo, L.-X., Yu, L.-Q., Zhang, Q., Xiong, J.-R., Zhu, X.-Y., Wang, X.-C., Huang, B.-C. & Jin, R.-C. Phosphorus removal from diluted wastewaters using a La/C nanocomposite–doped membrane with adsorption-filtration dual functions. *Chem. Eng. J.* **405**, 126924 (2021).
